# A network of RNA-binding proteins controls translation efficiency to activate anaerobic metabolism

**DOI:** 10.1038/s41467-020-16504-1

**Published:** 2020-05-29

**Authors:** J. J. David Ho, Nathan C. Balukoff, Phaedra R. Theodoridis, Miling Wang, Jonathan R. Krieger, Jonathan H. Schatz, Stephen Lee

**Affiliations:** 1 Department of Biochemistry and Molecular Biology, Miller School of Medicine, University of Miami, Miami, FL 33136 USA; 2 Sylvester Comprehensive Cancer Center, Miller School of Medicine, University of Miami, Miami, FL 33136 USA; 3 Division of Hematology, Department of Medicine, Miller School of Medicine, University of Miami, Miami, FL 33136 USA; 4 The SickKids Proteomics, Analytics, Robotics & Chemical Biology Centre (SPARC Biocentre), The Hospital for Sick Children, Toronto, ON M5G 1X8 Canada; 5 Department of Urology, Miller School of Medicine, University of Miami, Miami, 33136 USA; 6 Present Address: Bioinformatics Solutions Inc., Waterloo, ON N2L 6J2 Canada

**Keywords:** Translation, Regulatory networks

## Abstract

Protein expression evolves under greater evolutionary constraint than mRNA levels, and translation efficiency represents a primary determinant of protein levels during stimuli adaptation. This raises the question as to the translatome remodelers that titrate protein output from mRNA populations. Here, we uncover a network of RNA-binding proteins (RBPs) that enhances the translation efficiency of glycolytic proteins in cells responding to oxygen deprivation. A system-wide proteomic survey of translational engagement identifies a family of oxygen-regulated RBPs that functions as a switch of glycolytic intensity. Tandem mass tag-pulse SILAC (TMT-pSILAC) and RNA sequencing reveals that each RBP controls a unique but overlapping portfolio of hypoxic responsive proteins. These RBPs collaborate with the hypoxic protein synthesis apparatus, operating as a translation efficiency checkpoint that integrates upstream mRNA signals to activate anaerobic metabolism. This system allows anoxia-resistant animals and mammalian cells to initiate anaerobic glycolysis and survive hypoxia. We suggest that an oxygen-sensitive RBP cluster controls anaerobic metabolism to confer hypoxia tolerance.

## Abstract (web summary)

mRNA translation efficiency is regulated in response to stimuli. Here the authors employ mass spectrometry analysis of ribosome fractions and show that under hypoxia, oxygen-sensitive RNA binding proteins enhance the translation efficiency of glycolysis pathway transcripts.

## Introduction

Glycolysis is an ancient, core metabolic pathway conserved in all living organisms^1–3^, and its activation by the absence of oxygen is a central tenet of biology known as the Pasteur effect^4^. As a keystone of cellular energy production, anaerobic metabolism/glycolysis underlies hypoxia- and anoxia-tolerance across kingdoms^5–7^, from simple prokaryotes to complex eukaryotes including humans and hypoxia-resistant organisms e.g. naked mole rats^8^ and nematode worms^9^. Furthermore, hypoxia is a critical aspect of human health and disease^10,11^, where the inhibition of hypoxia-induced glycolysis compromises cellular survival^12^. Here, we address the fundamental question as to the conserved mechanisms that transduce oxygenic changes into glycolytic adaptations across the evolutionary spectrum.

There has been a recent revitalization of efforts in deciphering translational regulatory mechanisms, especially given that translatome remodeling and translation efficiency modifications often equal or predominate over transcript level fluctuations in controlling protein output^13–17^. This phenomenon has been observed during evolution^18^, development^19^, differentiation^20,21^, cell-type specificity^22^, circadian regulation^23^, and stress adaptation^14,24–31^. This raises the question as to the identity of translatome remodelers that target different mRNA populations to bring about global, stimuli-induced translatome reprogramming^25,32^, especially in response to physiological stimuli e.g., hypoxia^33–35^. Recent work indicates that adaptive translatome remodeling is driven by specialized translation machineries^36,37^, such as the hypoxic cap-binding complex eIF4F^H^ (consisting of eIF4E2^38^ and eIF4G3)^25,39^, eIF4E3^40^, eIF3d^37^, DAP5^41,42^, and eIF5B^43^. Yet, general translation factors *per se* possess only limited capabilities to discriminate between mRNAs. Thus, we hypothesized the existence of stimuli-adaptive translatome remodelers that collaborate with protein synthesis machineries to control the translation efficiencies of specific mRNA populations.

RBPs play a critical role in controlling various aspects of transcript fate and metabolism, including mRNA stability and translation efficiency^44^. In fact, RBP engineering represents a significant advancement in the development of programmable therapeutics involving synthetic RNA/translation-based circuits for a myriad of diseases^45,46^. The dynamic relationship between mRNAs and RBPs is extremely complex. Vital earlier studies demonstrated the existence and mechanisms of these relationships using model RBPs or mRNAs^47–50^. Studies that identify cellular repertoires of RBP/mRNA interactions, have been critical for the characterization of RBP identity^51–54^. Here, we introduce an unbiased system-wide investigation of RBP translational engagement using the MATRIX platform^43^, followed by global translatome analyses using TMT-pSILAC to determine the proteins and cellular pathways regulated by hypoxia-adaptive RBPs.

In this study, we report an oxygen-sensitive cluster of RBPs that controls the translation efficiency of mRNAs encoding proteins that effect anaerobic metabolism. Disruption of this network renders mammalian cells and the anoxia-tolerant *C. elegans* sensitive to mild hypoxia by preventing anaerobic glycolysis. This RBP system collaborates with the recently characterized hypoxic translation machinery^25,39,43^, providing a potential explanation for the switch to anaerobic metabolism that confers hypoxia tolerance across species.

## Results

### System-wide profile of oxygen-responsive translational RBPs

**Fig. 1:**
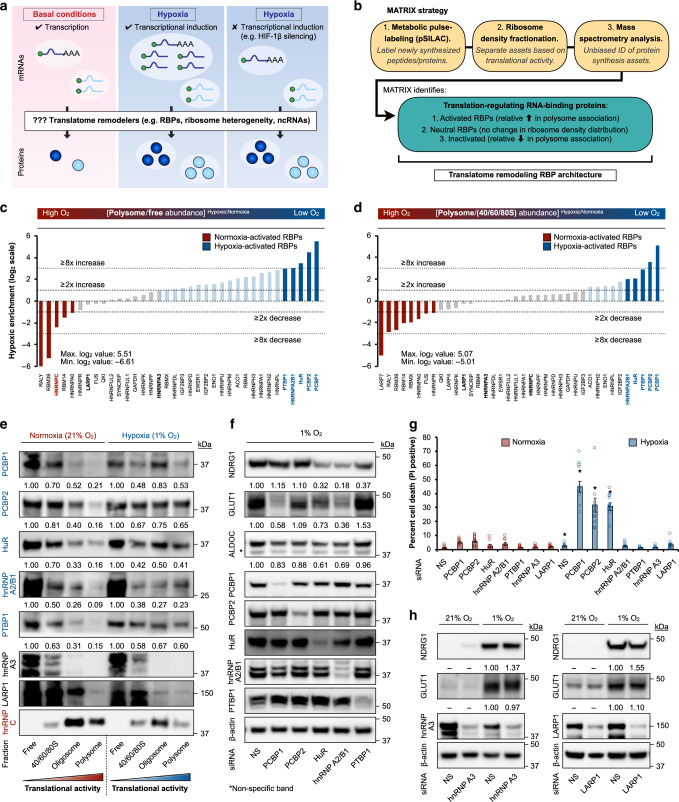
Oxygen-sensitive rewiring of RBP engagement in protein synthesis. **a** Model of hypoxia-induced translatome remodeling, highlighting the concept that translational reprogramming resulting in increased protein synthesis can occur independently of mRNA-level adaptations. Color scheme: red, normoxia; blue, hypoxia. **b** Schematic of MATRIX platform. Asset refers to proteins involved in translation, e.g., translation factors, ribosomal proteins, RBPs, etc. **c** Primary MATRIX readout of relative RBP translational engagement (ratio of polysome/free protein abundance) in hypoxic (1% O_2_, 24 h, blue) versus normoxic (21% O_2_, 24 h, red) U87MG. **d** Secondary MATRIX readout of relative RBP translational engagement (ratio of polysome/monosome protein abundance). Validated RBPs (**e**) are bolded. **e** Representative immunoblots of indicated RBPs in normoxic and hypoxic U87MG ribosome density fractions. RBPs whose translational activity is activated and repressed by hypoxia are highlighted in blue and red, respectively. Quantitation represents mean of three independent experiments (*n* = 3). **f** Representative immunoblots of U87MG treated with siRNAs (for 48 h prior to following experimentation) against hypoxia-adaptive RBPs. NS: non-silencing. Quantitation represents mean of four independent experiments (*n* = 4). **g** Cell death measurements by propidium iodide (PI) staining in U87MG treated with indicated siRNAs. Asterisk denotes statistical significance calculated using two-sided Student’s *t*-tests compared to corresponding normoxic measurements. Exact *p* values: NS siRNA (*p* = 0.005), PCBP1 siRNA (*p* = 2.11e−06), HuR siRNA (*p* = 0.0006), and hnRNP A2/B1 siRNA (*p* = 2.08e−07). Data represent mean ± SEM (error bars) (*n* = 10 fields over three independent experiments). **h** Representative immunoblots of U87MG treated with siRNAs against non-hypoxia-activated RBPs. NS: non-silencing. Quantitation represents mean of three independent experiments (*n* = 3). Source data are provided as a Source data file.

Here, we address the question as to how cellular pathways are regulated in response to stimuli via translatome remodeling. We focused on identifying translatome remodelers that select and modify the translatability of pre-existing and newly synthesized mRNAs, using the physiological stress of hypoxia as a model (Fig. 1a)^25,55–57^. RBPs control mRNA stability and translation efficiency, serving as critical rheostats of protein expression during stimuli responses^44^. We performed a global, impartial screen using our recently developed MATRIX (mass spectrometry analysis of active translation factors using ribosome density fractionation and isotopic labeling experiments) technology (Fig. 1b, Supplementary Fig. 1a)^43^ to generate an oxygen-responsive, activity-based blueprint of RBP translational utilization (enabled by ribosome density fractionation) (Supplementary Fig. 1b). In general, polysome fractions contain factors and mRNAs undergoing intense, productive translation. In contrast, free fractions are relatively enriched for factors that are disengaged from active protein synthesis, while the 40/60/80/S monosome fractions allow for a more focused assessment of factors involved in translation initiation. Metabolic pulse-labeling with SILAC (pSILAC) enables the labeling and minimization of confounding signals from newly synthesized peptides. Specifically, pulse-labeling with heavy isotopes (R_10_K_8_) preferentially labels de novo synthesized peptides over existing translation machinery components (Supplementary Fig. 1a). Heavy SILAC signals are excluded from the downstream analysis to allow a clearer focus on the abundance of machinery components. In this study, we focused on the RBPs that displayed the largest degree of hypoxic activation in terms of translational engagement (primarily translation elongation) compared to cells maintained in normoxia. Using the ratio of polysome/free protein abundance as a primary readout and the ratio of polysome/monosome protein abundance as a secondary readout, results indicated that translational activities of PCBP1, PCBP2, HuR (ELAVL1), hnRNP A2/B1, and PTBP1 were prominently increased under hypoxic conditions (Fig. 1c, d), in contrast to others that exhibited no change (e.g., hnRNP A3, LARP1) or reduced (e.g., hnRNP C) translational engagement. The polysome/free ratio was used as the primary measure as it reflects translational involvement (i.e. intense, productive translation versus translationally inactive fractions). Factors engaged in monosomes could be interpreted as promoters of translation initiation. However, monosomes can also be enriched for nonsense-mediated decay protein factors and target transcripts^58^. Thus, to minimize potential confounding signals from monosome fractions, we used the polysome/monosome ratio as a secondary readout. We note that RBPs represent a sub-population of polysome-engaged proteins, which also includes proteins that interact indirectly with RNA. The ribosome density distribution of representative RBPs (bolded in Fig. 1c, d) were confirmed by immunoblots of ribosome density fractions (Fig. 1e, Supplementary Fig. 1c), and were not due to changes in steady-state protein levels (Supplementary Fig. 1d, e). This platform also confirmed our previous finding of RBM4 as a hypoxia-adaptive RBP^39^ (Fig. 1c, d). Consistent with MATRIX analysis and immunoblot validations using ribosome density fractions which demonstrated enhanced hypoxic translational engagement, we observed a hypoxia-associated increase in cytoplasmic localization for PCBP1, hnRNP A2B1, and HuR (Supplementary Fig. 1f, g). Analysis of monosome/free abundances suggest that these RBPs are more heavily involved in translational elongation (Fig. 1c, d) compared to initiation (Supplementary Fig. 1h). We focused this current study on the five most highly activated RBPs by hypoxia from a population of confidently detected proteins, while future studies will examine other RBPs identified by MATRIX. These results led us to hypothesize that hypoxia-adaptive RBPs form a dynamic collaborative network to drive oxygen-responsive translational adaptations.

### O_2_-sensitive RBPs control hypoxic adaptation and survival

Confirming the biological relevance of this RBP network to hypoxic adaptation, we found that the individual silencing of these RBPs significantly attenuated the induction of classic hypoxia-inducible proteins e.g. NDRG1, GLUT1, and ALDOC (Fig. 1f, Supplementary Fig. 1i), each of which were regulated by several RBPs. This phenomenon was confirmed in additional human cell lines (Supplementary Fig. 2a, b). We point out that relatively small but reproducible magnitudes of change e.g., for ALDOC can be expected, as most mRNAs can be concurrently regulated by an array of RBPs that may exert collaborative or competing effects. Likewise, even though PCBP2 silencing did not appear to alter the steady-state protein levels of the three representative targets shown, this does not preclude its role in regulating other hypoxia-inducible proteins/pathways. Notably, cell death (Fig. 1g, Supplementary Fig. 2c, d) and cell viability (Supplementary Fig. 2e) measurements revealed a significantly greater effect of hypoxia-adaptive/oxygen-sensitive RBPs e.g. PCBP1, HuR, and hnRNP A2/B1 on hypoxic cellular survival compared to normoxic conditions. These effects on cell viability measured after 72 h of hypoxic treatment were also observed at 24 h of hypoxic treatment (Supplementary Fig. 2f, g). Supporting the specificity of these effects by hypoxia-adaptive RBPs, RBPs not activated by hypoxia (e.g., hnRNP A3, LARP1) did not display differential effects on cell death or viability in response to oxygenic changes (Fig. 1g, Supplementary Fig. 2c–e, g), or affect the expression of hypoxia-inducible targets (Fig. 1h, Supplementary Fig. 2h). Corroborating the siRNA-mediated silencing experiments, treatment with the HuR-inhibitor CMLD-2^59^ attenuated NDRG1 and GLUT1 induction in multiple human cell lines and mouse embryonic fibroblasts (Supplementary Fig. 2i, j). Taken together, these findings establish the adaptive significance of hypoxia-adaptive RBPs.

### Hypoxia-adaptive RBPs control translatome reprogramming

**Fig. 2:**
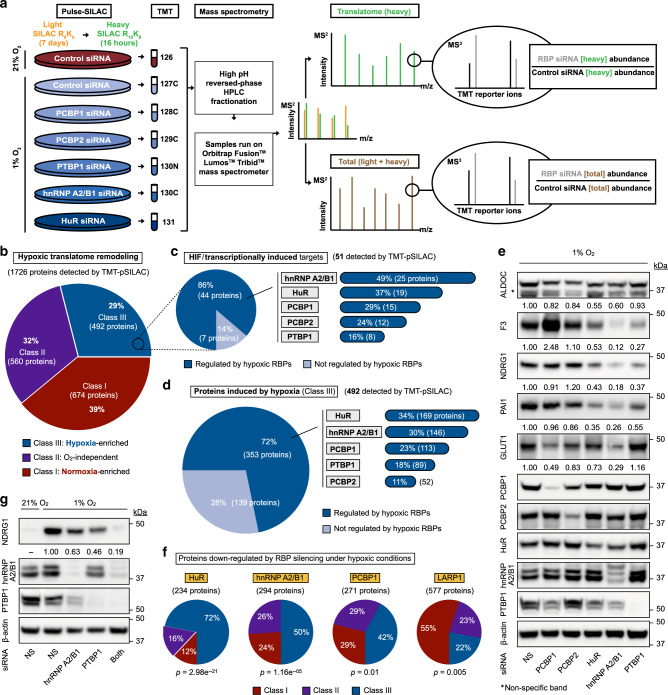
Translatome (protein output) remodeling by hypoxia-adaptive RBPs. **a** Schematic of TMT-pSILAC translatome analysis strategy. Color scheme: red, normoxia; blue, hypoxia. **b** Hypoxic translatome (protein output) remodeling. Class III: hypoxia-enriched proteins (at least 15% increase protein output under hypoxic conditions); Class II: oxygen-neutral proteins (no change in protein output); Class I: normoxia-enriched proteins (at least 15% decrease in protein output under hypoxic conditions). **c** Classical HIF/transcriptionally induced targets represent ~10% of all Class III proteins. Left panel: of these, 86% are regulated by the top five hypoxia-adaptive RBPs identified by MATRIX. Right panel: percentage and number of proteins regulated by each RBP. **d** Left panel: the majority (72%) of all Class III, hypoxia-inducible proteins are regulated by the top five hypoxia-adaptive RBPs identified by MATRIX. Right panel: percentage and number of proteins regulated by each RBP. **e** Representative immunoblots of U87MG treated with siRNAs (for 48 h prior to following experimentation) against hypoxia-adaptive RBPs. NS: non-silencing. Quantitation represents mean of three independent experiments (*n* = 3). **f** Classification of proteins downregulated at the production level by knockdowns of indicated RBPs. *p* values represent Chi-Square tests for significant proportional differences compared to overall hypoxic translatome remodeling (presented in **b**). **g** Representative immunoblots of U87MG treated with siRNAs against one or two hypoxia-adaptive RBPs. NS: non-silencing. Quantitation represents mean of three independent experiments (*n* = 3). Source data are provided as a  Source data file.

Next, we analyzed the global RBP-dependent translatomes (protein outputs) using tandem mass tag-pulse SILAC (TMT-pSILAC) and mass spectrometry (MS) for each of the top five hypoxia-adaptive RBPs under hypoxic and normoxic conditions, as well as the non-hypoxia-activated RBP LARP1 as a control (Fig. 2a, Supplementary Fig. 3a, b). Systemic translatome reorganization is a cornerstone of hypoxic adaptation, with ~30% of the cellular mRNA population exhibiting hypoxia-enhanced translation efficiency and protein output (i.e., Class III members)^25^. Our current analysis recapitulated this phenomenon, identifying 492 hypoxia-inducible (Class III) proteins (Fig. 2b). Of these, ~10% (51 proteins) corresponded to documented HIF transcriptional targets whose mRNA production is augmented under hypoxic conditions (Fig. 2c). Given differences in treatment conditions, cell type differences, and experimental approach, there is currently no firm consensus on the number of HIF targets. As such, we note that there may be additional, yet unidentified/unconfirmed HIF targets in the Class III population. Notably, protein production of the majority (84%) of these targets are dependent on one or more hypoxia-adaptive RBPs (Fig. 2c, Supplementary Table 1), whereby the silencing of one or more RBPs led to a decrease in the synthesis of these proteins. Expanding upon this analysis, results indicated that 72% of the entire hypoxia-inducible protein population, many of which do not exhibit significant hypoxia-induced changes in mRNA levels^25^, are dependent on the top five hypoxia-adaptive RBPs for efficient protein synthesis (Fig. 2d). Advantages of TMT-pSILAC multiplexing include the reduction of inter-run variability and enhanced precision, quantitative power, and the ability to identify significant regulations^60,61^. A minor caveat of such quantitative multiplexing is a relatively compressed range of sensitivity^62^. As such, we validated our omic regulatory threshold (0.15x difference) using targeted measurements of steady-state protein expression (Fig. 2e, Supplementary Fig. 3c). The relative effect of each RBP on protein output was qualitatively reproduced by translatomic and steady-state proteomic observations (Fig. 1f, Supplementary Fig. 1i, 2e, Supplementary Fig. 3c, d). In addition, analysis revealed strong concordance and coefficients of determination between protein output and steady-state protein levels (Supplementary Fig. 3e,f), supporting translational regulation as a contributor to overall changes in protein levels during cellular stress responses, in addition to other potential adaptations e.g., steady-state mRNA level changes. Further analyses showed that the majority of proteins downregulated by the silencing of HuR, hnRNP A2/B1, and PCBP1 belonged to the hypoxia-inducible Class III category (Fig. 2f). In contrast, this was not observed for non-hypoxia-activated RBPs e.g., LARP1 (Fig. 2f). We note that even though TMT-pSILAC measures changes in protein synthesis/output, we acknowledge the potential contributions of protein degradation toward changes in overall steady-state protein expression. The enrichment of hypoxia-inducible proteins in the RBP-dependent target population compared to overall hypoxic protein production (Fig. 2b) supports the selectivity of hypoxia-activated RBPs for hypoxia-adaptive proteins. In support of synergy between RBPs, we observed a greater effect of hnRNP A2/B1 and PTBP1 double-silencing on steady-state NDRG1 protein expression (Fig. 2g, Supplementary Fig. 3g). Together, these findings establish hypoxic translatome remodeling as a major function of oxygen-sensitive RBPs.

### Anaerobic glycolysis is controlled by hypoxia-adaptive RBPs

**Fig. 3:**
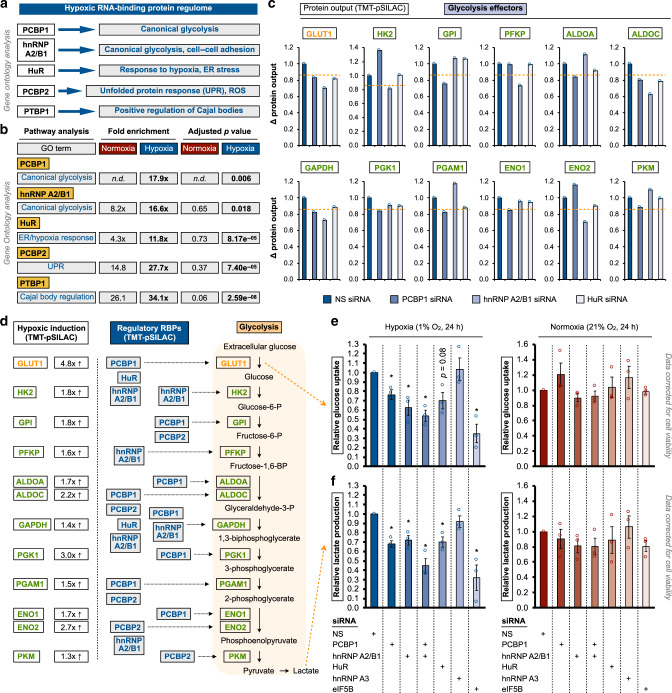
Oxygen-dependent reconfiguration of RBP-regulated cellular networks. **a** Hypoxia-induced rewiring of RBP-regulated processes by Gene Ontology (GO) pathway enrichment analysis. **b** GO analysis of representative enriched biological processes for downregulated proteins when indicated RBPs are silenced. **c** Protein output regulation of each glycolytic effector by the hypoxia-adaptive RBPs PCBP1, hnRNP A2/B1, and HuR, as determined by TMT-pSILAC (three independent experiments pooled into a single sample for measurement). Orange dotted line represents the MS regulatory threshold, validated empirically at the steady-state protein level by immunoblot, and at the translation efficiency level by qRT-PCR of ribosome density fractions. **d** Left panel: protein expression of glycolytic effectors are induced under hypoxic conditions (confirmed by TMT-pSILAC). Synergistic regulation of glycolytic proteins (right panel) by hypoxia-adaptive RBPs (middle panel) as determined by TMT-pSILAC analysis. Measurements of **e** glucose uptake and **f** lactate production in U87MG treated with indicated siRNAs (for 48 h prior to following experimentation). Color scheme: red, normoxia; blue, hypoxia. NS: non-silencing. Asterisk denotes statistical significance calculated using two-sided Student’s *t*-tests compared to NS control. Exact *p* values **e**: PCBP1 siRNA (*p* = 0.04), hnRNP A2/B1 siRNA (*p* = 0.04), PCBP1 + hnRNPA2/B1 siRNA (*p* = 0.02), eIF5B siRNA (*p* = 0.02). Exact *p* values **f**: PCBP1 siRNA (*p* = 0.01), hnRNP A2/B1 siRNA (*p* = 0.03), PCBP1 + hnRNPA2/B1 siRNA (*p* = 0.02), HuR siRNA: (*p* = 0.03), eIF5B siRNA (*p* = 0.04). Data represent mean ± SEM (error bars) of three independent experiments. Source data are provided as a Source data file.

TMT-pSILAC and pathway analyses revealed that upon exposure to hypoxia, hnRNP A2/B1, and PCBP1 are re-tasked to activate anaerobic glycolysis (Fig. 3a, b, Supplementary Fig. 4a, b). A closer examination of TMT-pSILAC results indicated that a number of glycolytic effectors are regulated by hypoxia-adaptive RBPs (Fig. 3c, Supplementary Fig. 4c). In particular, PCBP1 and hnRNP A2/B1 regulated eight and six glycolytic proteins, respectively, which are well-established hypoxia-inducible proteins (Fig. 3c, d)^63–66^. Analysis of CLIP datasets^67–70^ provided evidence that hypoxia-activated RBPs can engage in direct interactions with the mRNAs of glycolytic effectors (Supplementary Table 2), confirming their established roles as RNA-binding proteins^47–54^. In contrast, non-hypoxia-activated RBPs e.g., LARP1 affected only two glycolytic enzymes (Supplementary Fig. 4c). Validating the functional relevance of these findings, individual and combined silencing of PCBP1, hnRNP A2/B1, or HuR significantly reduced glucose uptake (Fig. 3e) and lactate production (Fig. 3f) specifically under hypoxic, but not normoxic conditions (Fig. 3e, f). This effect was confirmed under prolonged hypoxic conditions (72 h), which was performed to allow cells to acclimatize and reach a new metabolic steady-state (Supplementary Fig. 4d, e). Induction of glycolytic effector protein levels are comparable between 24 and 72 h of hypoxic treatment (Supplementary Fig. 4f, g). Knockdown of the translation factor eIF5B was included as a positive control (Fig. 3e, f, Supplementary Fig. 4d, e), as we recently demonstrated its requirement for hypoxic glycolytic activation^43^. As an additional control for specificity, the non-hypoxia-activated RBP hnRNP A3 did not affect anaerobic glycolysis (Fig. 3e, f, Supplementary Fig. 4d, e). Together, these findings suggest an oxygen-regulated switch in RBP-dependent processes, and demonstrate the role of hypoxia-adaptive RBPs in activating anaerobic glycolysis.

### Hypoxic RBPs control glycolytic mRNA translation efficiency

**Fig. 4:**
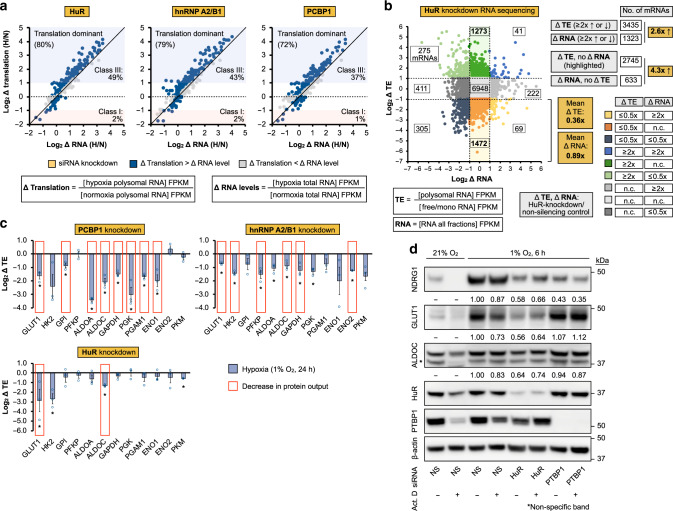
Hypoxia-adaptive RBPs promote glycolytic induction through translation efficiency. **a** Mechanism of hypoxic induction for RBP-dependent translatomes. Global analysis of hypoxia-induced translational versus RNA-level changes in U87MG using RNA sequencing of ribosome density fractions for HuR-, hnRNP A2/B1-, and PCBP1-dependent targets. Color scheme: red, Class I proteins, normoxia-enriched; blue, Class III proteins, hypoxia-enriched. **b** Global analysis of HuR-dependent changes in mRNA translation efficiency and steady-state expression in U87MG using RNA sequencing of ribosome density fractions following siRNA-mediated HuR-silencing. **c** Hypoxic translation efficiency regulation of glycolytic effectors by the hypoxia-adaptive RBPs PCBP1, hnRNP A2/B1, and HuR, as determined by qRT-PCR of ribosome density fractions. Asterisk denotes statistical significance calculated using two-sided Student’s *t*-tests compared to non-silencing control. Exact *p* values: PCBP1 siRNA: GLUT1 (*p* = 0.02), GPI (*p* = 0.03), ALDOA (*p* = 0.001), ALDOC (*p* = 0.02), GAPDH (*p* = 0.007), PGK (*p* = 0.03), PGAM1 (*p* = 0.004), ENO1 (*p* = 0.047); hnRNP A2/B1 siRNA: GLUT1 (*p* = 0.007), HK2 (*p* = 0.004), PFKP (*p* = 0.04), ALDOA (*p* = 0.04), ALDOC (*p* = 0.048), GAPDH (*p* = 0.05), PGK (*p* = 0.03), ENO2 (*p* = 6.79e−05); HuR siRNA: GLUT1 (*p* = 0.04), HK2 (*p* = 0.04), ALDOC (*p* = 0.001), PKM (*p* = 0.0008). Data represent mean ± SEM (error bars) of three independent experiments (*n* = 3). Red box: observed decrease in protein output as determined by TMT-pSILAC. **d** Representative immunoblots of normoxic and hypoxic (6 h) U87MG treated with actinomycin D (+) or vehicle DMSO (−). NS: non-silencing. Quantitation represents mean of three independent experiments (*n* = 3). Source data are provided as a Source data file.

RBPs have well-established roles in regulating mRNA translation efficiency and stability, two intricately linked aspects of mRNA metabolism that affect protein output^44,47–49^. We defined translation efficiency as the ratio of mRNA abundance in polysome fractions (intense protein synthesis) to mRNA abundance in free/monosome fractions (no/little protein synthesis)^25,43^. Steady-state mRNA expression is defined as the aggregate abundance across all fractions^25,43^. To elucidate the relative effects of hypoxic RBPs on target translational intensity versus RNA levels, we first established that targets of hypoxia-activated RBPs are predominantly regulated at the translational level in response to oxygen deficiency (Fig. 4a, Supplementary Fig. 5a–c). This finding confirms our recent report that increased translation efficiency is a primary mechanism by which hypoxia-inducible (i.e., Class III) proteins accumulate^25^, and suggested that hypoxic RBPs regulate protein output at least in part through translation efficiency modifications. Testing this hypothesis, we performed an RNomic analysis of global translation efficiency and RNA level modifications in response to RBP depletion during hypoxia, using HuR as a prototypical model since it regulates the largest proportion of Class III proteins (Fig. 2d, Supplementary Fig. 5d, e). First, ribosome density profiling revealed that hypoxic HuR silencing resulted in a general reduction in translation efficiency, as demonstrated by a decrease in the proportion (area under the curve) of polysomes (≥5 ribosomes; high-intensity protein synthesis) compared to non-silencing control (Supplementary Fig. 5f). RNomic analysis indicated that overall, HuR depletion affected ~three times as many mRNAs at the translation efficiency level (3435) compared to RNA level changes (1323) (based on a two-fold difference threshold) (Fig. 4b). In particular, HuR regulates a prominent population of transcripts at the translation efficiency, but not RNA level (2745), which was ~four times the number of targets affected at the RNA, but not translation efficiency level (633) (Fig. 4b). We also observed that the average magnitude of translation efficiency decrease was higher than that of RNA level reductions, especially for those transcripts downregulated at the translation efficiency level by HuR silencing (64% decrease at the translation efficiency level versus 11% at the RNA level) (Fig. 4b). Translational regulation by HuR often depends on its interactions with AU-rich elements (AREs) on the 3′-UTRs of target mRNAs^71–73^. As expected, bioinformatic analysis confirmed an overall enrichment of AREs in the 3′-UTRs of mRNAs regulated by HuR (translational and mRNA expression) compared to the total mRNA population in our system (Supplementary Fig. 5g). In particular, translationally repressed targets of HuR (up-regulated by HuR knockdown) exhibited a ~two-fold enrichment of an established mRNA-destabilizing ARE motif^74^ compared to translationally enhanced targets of HuR (Supplementary Fig. 5g).

Next, we performed targeted validations of translation efficiency regulation for each glycolysis effector. Specifically, qRT-PCR measurements confirmed potent RBP-dependent effects on the translation efficiencies of glycolysis proteins (Fig. 4c, Supplementary Fig. 5h), as well as VEGFA, a well-established post-transcriptional target of HuR we employed as a control (Supplementary Fig. 5i)^48,75^. Notably, RBP-dependent changes in translation efficiency closely matched protein level measurements by TMT-pSILAC, representing a more accurate predictor and prominent contributor to protein output compared to steady-state RNA level alterations (Fig. 4c, Supplementary Fig. 5j, k). Furthermore, we demonstrated the independence of RBP-mediated translation efficiency adaptation from mRNA level fluctuations using actinomycin D treatment, which suppressed mRNA induction yet did not affect RBP-mediated protein inductions (Fig. 4d, Supplementary Fig. 5l, m). These results suggest that RBPs function as a downstream checkpoint of protein output for both existing and newly synthesized mRNAs. Together, these findings demonstrate that while hypoxia-adaptive RBPs regulate protein output synergistically via both translation efficiency and transcript level changes, translation efficiency regulation can represent the predominant mechanism of translatome remodeling.

### Hypoxic RBPs collaborate with hypoxic translation machinery

**Fig. 5:**
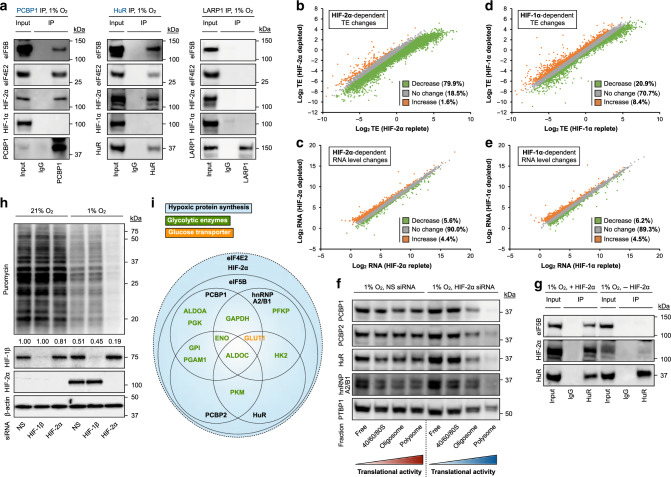
Oxygen-sensitive RBPs collaborate with the hypoxic protein synthesis machinery. **a** Representative immunoblots of HuR, PCBP1 (hypoxia-adaptive, blue), and LARP1 (non-hypoxia-activated, black) co-immunoprecipitations in U87MG. Three independent experiments (*n* = 3) were performed with similar results. Global RNA sequencing analysis of (**b**, **c**) HIF-2α- and (**d**, **e**) HIF-1α-dependent changes in (**b**, **d**) translation efficiency (TE) and (**c**, **e**) steady-state mRNA levels. **f** Representative immunoblots of hypoxia-adaptive RBPs in hypoxic U87MG ribosome density fractions with and without HIF-2α siRNA-mediated knockdown (for 48 h prior to following experimentation). Color scheme: red, normoxia; blue, hypoxia. Three independent experiments (*n* = 3) were performed with similar results. **g** Representative immunoblots of HuR co-immunoprecipitations with and without HIF-2α siRNA-mediated knockdown in U87MG. Three independent experiments (*n* = 3) were performed with similar results. **h** Representative immunoblots of U87MG treated with indicated siRNAs. Puromycin incorporation was used as a measure of global translational intensity. NS: non-silencing. Quantitation represents mean of three independent experiments (*n* = 3). **i** Empirically derived model of functional integration between hypoxia-adaptive RBPs and elements of the hypoxic protein synthesis machinery. Source data are provided as a Source data file.

As gatekeepers of mRNA translation efficiency (Fig. 4), RBPs cooperate closely with the translational apparatus to regulate protein production^76,77^. For instance, RBM4^39^, which was independently confirmed by MATRIX (Fig. 1c), assembles with the hypoxic protein synthesis machinery. We recently discovered that the ancient translation factor eIF5B functions as a hypoxic surrogate of eIF2, enabling initiator methionine-tRNA (met-tRNAi^Met^) delivery during oxygen deficiency^43^. Notably, central carbon metabolism, including anaerobic glycolysis, was identified as a prominent dependent of hypoxic eIF5B activity. This explains the dramatic decrease in glycolytic intensity in hypoxic, eIF5B-deficient cells (Fig. 3e, f, Supplementary Fig. 4d. e). Consistent with these findings, we confirmed that hypoxia-activated RBPs e.g., PCBP1 and HuR associate with elements of the hypoxic protein synthesis machinery, including eIF5B and the hypoxic cap-binding protein eIF4E2 (Fig. 5a, left and middle panels). In contrast, such interactions were not observed for non-hypoxia-activated RBPs e.g., LARP1 (Fig. 5a, right panel). The hypoxic translational apparatus contains hypoxia-inducible factor 2α (HIF-2α) as an oxygen-sensing activator^39,78^. In line with this, HIF-2α, but not the closely related isoform HIF-1α, co-immunoprecipitated with hypoxic RBPs HuR and PCBP1 (Fig. 5a). Reciprocal pull-down experiments confirmed the association between HIF-2α (but not HIF-1α) and hypoxia-activated RBPs PCBP1, hnRNP A2/B1, HuR, and PTBP1 (Supplementary Fig. 6a, b). Providing further support for the specificity of these relationships, non-hypoxia-activated RBPs hnRNP A3 and LARP1 did not co-immunoprecipitate with HIF-2α (Supplementary Fig. 6a). Furthermore, RNomics analyses (Supplementary Fig. 6c, d) revealed that HIF-2α exerted a prominent global effect on hypoxic translational intensity (Supplementary Fig. 6e, f) and mRNA translation efficiency (Fig. 5b), with a relatively minor effect on steady-state mRNA levels (Fig. 5c). In contrast, HIF-1α demonstrated a much lower effect on global protein synthesis (Supplementary Fig. 6e, g) and translation efficiency (Fig. 5d), while exhibiting a similar effect on mRNA levels (Fig. 5e). In line with its role in protein synthesis, HIF-2α, but not HIF-1α, was abundantly cytoplasmic (Supplementary Fig. 6h) and associated with hypoxic translating ribosomes (Supplementary Fig. 6i). HIF-2α depletion reduces the association of hypoxic RBPs with translating ribosomes (Fig. 5f, Supplementary Fig. 6j), at least in part by diminishing their ability to assemble with hypoxic translation factors e.g., eIF5B (Fig. 5g). We note that the effect of HIF-2α on hypoxic protein synthesis is independent of its transcriptional activity (Fig. 5h, Supplementary Fig. 6k), as previously reported^25,39^. Data from this study (RBP, HIF-2α analyses) and recently reported eIF5B and eIF4F^H^ results^35,46,50^ reveals a model of functional synergy between the hypoxia-adaptive RBP network and the hypoxic translation machinery in controlling the mRNA translation efficiencies of glycolytic enzymes and effectors (Fig. 5i).

### Conservation of glycolytic control by oxygen-sensitive RBPs

**Fig. 6:**
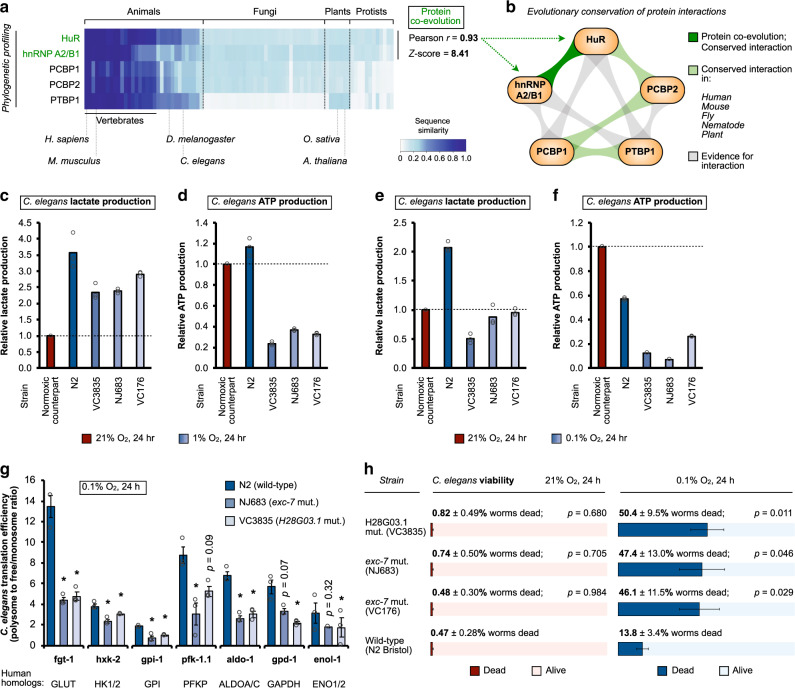
The oxygen-sensitive RBP network regulates hypoxia sensitivity across species. **a** Protein sequence similarity of hypoxia-activated RBPs across species. High protein sequence similarity (compared to the human homolog, first column) is indicated by dark blue coloring, and low protein similarity by light blue/white coloring (see legend on bottom right corner of panel). Analysis using the normalized phylogenetic profiling algorithm identified evidence suggesting co-evolution between HuR and hnRNP A2/B1 (Pearson *r* = 0.93, z score = 8.41). **b** Analysis of published RBP interactome data using the BioGRID bioinformatics resource reveals conserved protein interactions (indicated in green) between hypoxia-adaptive RBPs. An interaction is considered conserved if observed in human, mouse, fly, nematode, and at least one plant species. Measurements of **c**, **e** lactate and **d**, **f** ATP production in *C. elegans* subjected to **c**, **d** 1% O_2_ or **e**, **f** 0.1% O_2_. Color scheme: red, normoxia; blue, hypoxia. Data represent mean of triplicate measurements from a representative experiment. Three independent experiments (*n* = 3) were performed with similar results. **g** Translation efficiencies of glycolytic effectors in hypoxic (0.1% O_2_) *C. elegans* that contain wild-type (N2) or mutant homologs of HuR (*exc-7*, strain NJ683) or hnRNP A2/B1 (H28G03.1, strain VC3835), as determined by qRT-PCR of ribosome density fractions. Asterisk denotes statistical significance calculated using two-sided Student’s *t*-tests compared to wild-type control. Exact *p* values: NJ683: fgt-1 (*p* = 0.02), hxk-2 (*p* = 0.04), gpi-1 (*p* = 0.02), pfk-1.1 (*p* = 0.03), aldo-1 (*p* = 0.002); VC3835: fgt-1 (*p* = 0.03), hxk-2 (*p* = 0.04), gpi-1 (*p* = 0.003), aldo-1 (*p* = 0.009), gpd-1 (*p* = 0.02), enol-1 (*p* = 0.004). Data represent mean ± SEM (error bars) of three independent experiments (*n* = 3). **h** Measurements of normoxic and hypoxic (0.1% O_2_) death for adult (day 1 at the start of treatment) *C. elegans* that contain wild-type (N2) or mutant homologs of HuR (strains NJ683 and VC176) or hnRNP A2/B1 (strain VC3835). Statistical significance was calculated using two-sided Student’s *t*-tests compared to wild-type control. Exact *p* values are indicated in the figure. Data represent mean ± SEM (error bars) of four independent experiments (*n* = 4; total number of worms for N2, NJ683, VC176, VC3835: 927, 860, 753, 731, respectively). Source data are provided as a Source data file.

**Fig. 7:**
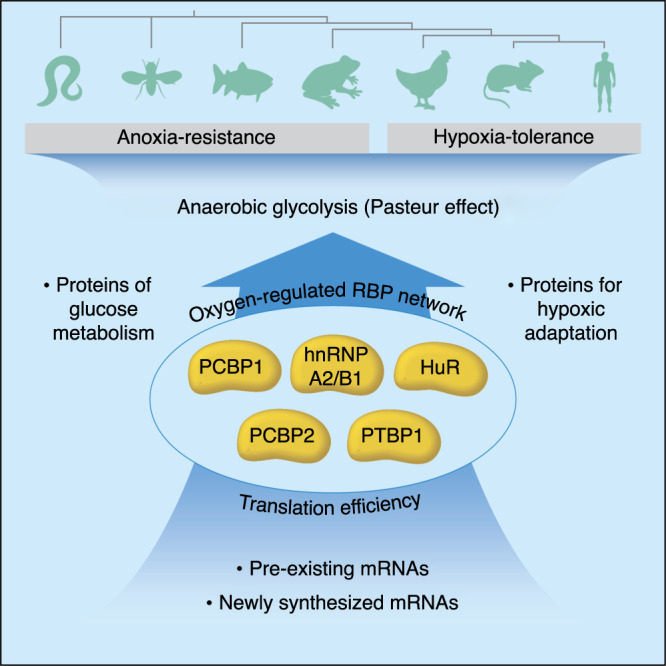
A network of RBPs activates anaerobic glycolysis through translation efficiency. An abstract representation of hypoxia-activated RBPs that activate anaerobic metabolism through mRNA translation efficiency of glycolytic proteins. This system enables hypoxic tolerance across species.

The glycolytic pathway is evolutionarily ancient; it is conserved in all living organisms, and likely evolved under anaerobic conditions in the last universal common ancestor^1–3,79–81^. Thus, we asked whether these oxygen-sensitive RBPs are conserved in their role of controlling glycolysis, especially in hypoxia-tolerant and/or resistant species e.g., *C. elegans*^9,82,83^. Protein co-evolution is an indicator of positive selection pressure to maintain interactions between proteins involved in key biological processes^84^. An algorithm (termed normalized phylogenetic profile) was recently established by the Ruvkun and Tabach laboratories to assess protein co-evolution by measuring sequence similarity while taking into account evolutionary distance^85–87^. Using this algorithm via the PhyloGene server^85^, we found evidence for protein co-evolution between the oxygen-sensitive RBPs HuR and hnRNP A2/B1 (Pearson correlation of 0.93, Z-score of 8.4) (Fig. 6a, Supplementary Fig. 7a). Analysis of published protein interactome datasets, including the use of the BioGRID database that contains annotated protein interactions across 66 organisms^88–90^, revealed conserved interactions between oxygen-sensitive RBPs, e.g., PCBP1/PCBP2, PCBP1/PTBP1, hnRNP A2/B1/HuR, and HuR/PCBP2 across species (Fig. 6b). Notably, *C. elegans* deletion mutants of hnRNP A2/B1 (H28G03.1)^91,92^ and HuR (*exc-7*) homologs (Supplementary Fig. 7b, c) demonstrated significantly reduced functional abilities to produce lactate and ATP under both 1% O_2_ (Fig. 6c, d) and 0.1% O_2_ (Fig. 6e, f) hypoxic conditions compared to their wild-type counterparts, recapitulating our observations in human cell lines (Fig. 3e, f). We prioritized 0.1% O_2_ for further *C. elegans* experiments because of the higher hypoxia tolerance of *C. elegans*, which naturally lives in hypoxic soil environments. In particular, 0.1% O_2_ elicits a robust hypoxia response, and has been used in multiple studies to examine hypoxic adaptation in *C. elegans*^93–96^. It has also been suggested to be in the range where oxygen concentration becomes significantly limiting for aerobic respiration in *C. elegans*^95^. Mechanistically, we confirmed decreased translation efficiencies of glycolytic effectors in these RBP mutants under hypoxic conditions (Fig. 6g) consistent with our mammalian observations (Fig. 4e). In contrast, total steady-state mRNA levels of glycolytic effectors remained unchanged in mutant strains versus their wild-type counterparts (Supplementary Fig. 7d), reminiscent of our mammalian observations (Supplementary Fig. 5h). Furthermore, we observed that while these RBP mutant strains were indistinguishable from their wild-type counterparts in terms of survival under standard growth conditions (Fig. 6h, left panel), the mortality of *exc-7* and H28G03.1 mutant *C. elegans* was markedly increased compared to their wild-type counterparts under hypoxic (0.1% O_2_) conditions (Fig. 6h, right panels). This in essence recapitulated our observations in mammalian cells (Fig. 1g, Supplementary Fig. 2c–g). As controls, *C. elegans* mutants for other *C. elegans* RBP paralogs either exhibited larval arrest under basal growth conditions (e.g., *hrp-1* strains VC406 and VC659, indicating additional basal/developmental functions, thereby precluding further analysis), or did not exhibit the same effect under hypoxic conditions e.g., *sqd-1* mutant strain VC1106 (Supplementary Fig. 7e). Consistent with these findings, and perhaps reflecting their decreased capacity to produce energy/fully activate glycolysis, *exc-7* mutants, but not wild-type controls, were reproducibly, albeit slightly, smaller (statistically significant difference of ~50 μm) under hypoxic conditions compared to their normoxic counterparts (Supplementary Fig. 7f). Taken together, these results suggest that oxygen-sensitive RBPs identified by MATRIX in mammalian cells have been conserved across species to regulate anaerobic metabolism and maintain hypoxic survival of anoxia-resistant animals (Fig. 7).

## Discussion

In the mid-19th Century, Louis Pasteur discovered that oxygen was a potent inhibitor of the glycolytic pathway. Here, we uncover a rate-limiting mechanism that explains the relationship between oxygen tension and the intensity of energy production by glycolysis. Using an unbiased MATRIX platform, we identified a cluster of oxygen-regulated RBP translatome remodelers that reprioritize the translation efficiencies of substantial mRNA populations as part of the hypoxic response. We show that these RBPs titrate proteins involved in glucose metabolism as a function of oxygen tension. These oxygen-regulated RBPs are essential for energy production by anaerobic glycolysis and viability in hypoxic mammalian cells as well as in anoxia-tolerant animals e.g., *C. elegans*. Phylogenetic analysis showed that oxygen-responsive RBPs are highly conserved in metazoans. Likewise, the oxygen-sensing HIF pathway and the hypoxic protein synthesis machinery are also conserved amongst multi-cellular organisms^43,97^.

We propose the following model that integrates this study with previous findings: Hypoxia-adaptive RBPs operate as downstream gatekeepers of the HIF/hypoxic transcriptional response^11,98^, increasing translation efficiencies of mRNAs encoding proteins of anaerobic metabolism by collaborating with the hypoxic translation machinery^25,39,43^. Depletion of hypoxia-adaptive RBPs prevents the induction of glycolytic effectors, effectively reducing the rate of glycolysis during hypoxia. We note that this working model does not preclude the participation of other translation pathways that may be operative in hypoxic cells, including eIF4E-dependent^99^ and cap-independent mechanisms e.g. IRES and CITE^100–102^. We also note that our current analyses are focused primarily on the involvement of RBPs in translation elongation (by virtue of polysomal engagement), and to a lesser degree translation initiation (monosomal engagement). Future studies will be required to examine the role of these and other RBPs in regulating other aspects of RNA metabolism e.g., splicing, nuclear-cytoplasmic export, and RNA degradation/stability. The RBP network that controls the ancient pathway of anaerobic glycolysis appears to be conserved, as its inactivation renders anoxia-resistant organisms (e.g., *C. elegans*) sensitive to hypoxia due to inhibited anaerobic glycolysis.

The evidence suggests that the regulation of mRNA translation efficiency by oxygen-sensitive RBPs dictate glycolytic protein levels and intensity, rather than simple fluctuations in mRNA level per se. The observation that a primordial pathway is controlled by a translational regulatory mechanism highlights the emerging paradigm that translational adaptations can sometimes supersede transcript-level changes^13,14^. We acknowledge, however, that under different biological conditions, other mechanisms of gene regulation may play an equal or more prominent role in determining steady-state protein levels, e.g., transcription-level changes^103^ and protein degradation/stability^104,105^. Nonetheless, from a broader perspective, our data may explain, at least in part, how protein levels are subject to stronger evolutionary pressure than mRNA levels^18,106^. While these RBPs control protein output via both translation efficiency and mRNA level modifications, our unbiased and targeted analyses suggest that the former appears to be the dominant mechanism, at least for certain RBP/mRNA interactions. It is possible that other RBPs may regulate translation predominantly through mRNA stability, and/or other aspects of mRNA processing e.g., splicing^107,108^. This study focused on the top five candidate RBPs from a confidently detected population identified by MATRIX that together control more than 70% of hypoxic protein induction. Nonetheless, we recognize the likely involvements other RBPs in hypoxic protein synthesis. For instance, the previously characterized RBM4 in hypoxic translation of growth factor receptors ranked 10^th^ in terms of hypoxic activation by MATRIX. Future studies will assess the roles of other oxygen-sensitive RBPs identified by MATRIX in hypoxic adaptation. We are cognizant of the hypomorphic nature of siRNA-based experiments and its potential influence on data interpretation, especially when multiple knockdowns are performed. In addition, we acknowledge that MATRIX may have missed lower abundance proteins/RBPs, which is an inherent caveat of data-dependent acquisition mass spectrometry. Finally, it is most likely that the full activation of anaerobic glycolysis (and other hypoxic response pathways) rely on the synergistic efforts of mRNA level inductions (e.g., by HIF) and translational augmentation by hypoxia-adaptive RBP networks.

Our current findings contribute to the growing body of evidence that underscores the critical role of RBPs in regulating cellular metabolism and hypoxic responses^47–50,109–116^. Complementing the classical approach of examining specific RBP–mRNA interactions, our study demonstrates the advantage of employing unbiased high throughput screens to identify stimuli/stress-sensitive RBPs based on biological activity. This systems-level approach allowed us to uncover a system of oxygen-responsive RBPs that facilitates the activation of anaerobic glycolysis/the Pasteur effect. Although the RBPs examined in this study are broadly expressed across cell types and tissues, we acknowledge the possibility that there may be cell type-dependent variations to this regulatory system.

Taken together, our evidence suggests that the activation of anaerobic metabolism is regulated by a conserved oxygen-responsive RBP network that controls the mRNA translation efficiencies of glycolytic proteins. It is likely that cells activate stimuli-adaptive RBP networks to control protein synthesis via translation efficiency to activate fundamental adaptive pathways in response to physiological stimuli. The challenge now will be to identify translation efficiency/translatome remodelers in the myriad fields of research that have been dominated by models of transcriptional regulation.

## Methods

### Cell culture and reagents

Human and mouse cell lines used in this study i.e., U87MG (Cat # HTB-14), PC3 (Cat # CRL-1435), A549 (Cat # CCL-185), HCT116 (Cat # CCL-247), and NIH/3T3 (Cat # CRL-1658) were purchased from the American Type Culture Collection (ATCC) and propagated in DMEM (HyClone) with 10 % FBS (Omega Scientific) and 1% penicillin-streptomycin (HyClone). Cells were maintained at 37 °C in a 5% CO_2_ humidified incubator. Cells were subjected to hypoxia (1% O_2_, 24 h, unless otherwise stated) at 37 °C in a 5% CO_2_, N_2_-balanced, humidified H35 HypOxystation (HypOxygen). CMLD-2 (Millipore Sigma) was used at final concentration of 25 μM for the last 24 h of treatment. Actinomycin D (Millipore Sigma) was used at final concentration of 1 μg/ml with 10 min pre-treatment before hypoxic or normoxic exposure. We note the possibility of off-target effects on observations made from drug treatment studies.

### *C. elegan*s culture and treatment

*C. elegans* strains were purchased from the Caenorhabditis Genetics Center (CGC), which is funded by NIH Office of Research Infrastructure Programs (P40 OD010440). Strains were maintained under standard conditions at 20 °C. N2 Bristol worms were utilized as the wild-type background. The following mutations and chromosome rearrangements were used: VC3835: H28G03.1(*gk3802*); NJ683: *exc-7*(*rh252*); VC176: *exc-7*(*ok370*); VC1106: *sqd-1*(ok1582). Hypoxic conditions were achieved and maintained in a humidified hypoxic chamber at 20 °C (Whitley DG250, Don Whitley Scientific) supplied with 0.1% or 1.0% O_2_ from dedicated tanks (5% CO_2_, N_2_-balanced, Airgas). Worms were synchronized by bleaching, and exposed to 0.1% O_2_ for 24 h starting as day 1 adults (lactate and ATP production measurements, translation efficiency and mRNA level measurements, and viability experiments), 1% O_2_ for 24 h starting as day 1 adults (lactate and ATP production experiments), and 1% O_2_ for 48 h starting as L1 larvae (body length measurements). Study protocols were approved by the University of Miami Institutional Animal Care & Use Committee.

### Pulse SILAC (pSILAC)

Cells were grown in light (R_0_K_0_) SILAC media (AthenaES) for 7 days and pulsed with heavy (R_10_K_8_) SILAC media (AthenaES) for 4 h (MATRIX) or 16 h (TMT-pSILAC) following treatment.

### Ribosome density fractionation

Polyribosome fractionations were performed based on our published protocols^43^. Briefly, cells were treated with 0.1 mg/ml of cycloheximide for the last 10 min of treatment, followed by ice-cold washes with PBS^−/−^ containing cycloheximide (0.1 mg/ml). Cells were then lysed in polysome lysis buffer (0.3 M NaCl, 15 mM MgCl_2_.6H_2_O, 15 mM Tris-HCl pH 7.4, 1% Triton X-100, 0.1 mg/ml cycloheximide, 100 units/ml RNase inhibitor). Following centrifugation (twice at 10,000 × *g* for 5 min at 4 °C) to remove cellular debris, samples were loaded based on equal total RNA onto a 10–50% sucrose gradient, and subjected to ultracentrifugation (187,813 × *g* for 1.5 h at 4 °C) using a SW 41 Ti rotor (Beckman Coulter). Samples were then fractionated into 1 ml fractions and collected using the BR-188 density gradient fractionation system (Brandel). Total RNA was isolated from each fraction by phenol-chloroform extraction and ethanol precipitation following proteinase K treatment. Total protein was isolated by TCA precipitation (20% final TCA concentration) followed by three ice-cold acetone washes. Three independent experiments were pooled into a single sample for MATRIX MS analysis.

### MS Analysis for MATRIX

Samples were resuspended in 100 μL of 50 mM NH_4_HCO_3_ (pH 8.3), 8 M Urea, and DTT was added to reduce cysteines at a final concentration of 10 mM. Cysteines were reduced at 60 °C for 1 h. Sample was cooled to room temperature and iodoacetamide was added to a final volume of 20 mM. Samples were incubated at room temperature in the dark for 30 min. Samples were then acetone precipitated overnight, and protein precipitates were centrifuged at 23,000 × *g* for 15 min. Precipitates were resuspended in 50 μL of NH_4_HCO_3_ (pH 8.3), and MS grade Trypsin/LysC (Promega) was added to a final protease:protein ratio of 1:50 and samples were digested overnight at 37 °C. Samples were lyophilized and resuspended in 0.1% trifluoroacetic acid (TFA). Peptides were fractionated using the Pierce High pH Reverse Phase Peptide Fractionation Kit (Pierce), following the manufacturer’s instructions. Each sample was fractionated into 8 high pH fractions.

Fractionated peptides were lyophilized, and lyophilized peptide mixtures were dissolved in 0.1% formic acid and loaded onto a 75 μm × 2 cm PepMap 100 Easy-Spray pre-column filled with 3 μm C18 beads (Thermo Fisher Scientific) followed by an in-line 75 μm × 50 cm PepMap RSLC EASY-Spray column filled with 2 μm C18 beads (Thermo Fisher Scientific) at a pressure of 700 BAR. Peptides were eluted over 120–240 min at a rate of 250 nl/min using a 0–35% acetonitrile gradient in 0.1% formic acid. For ribosome density fractionated samples, free fractions were eluted over 120 min each, while 40/60/80S and polysome fractions were eluted over 180 each. Peptides were introduced by nanoelectrospray into an LTQ-Orbitrap Elite hybrid mass spectrometer (Thermo-Fisher) outfitted with a nanospray source and EASY- nLC split-free nano-LC system (Thermo Fisher Scientific). The instrument method consisted of one MS full scan (400–1500 *m/z*) in the Orbitrap mass analyzer, an automatic gain control target of 1e6 with a maximum ion injection of 200 ms, one microscan, and a resolution of 240,000. Ten data-dependent MS/MS scans were performed in the linear ion trap using the ten most intense ions at a normalized collision energy of 35. The MS and MS/MS scans were obtained in parallel fashion. In MS/MS mode automatic gain control targets were 1e5 with a maximum ion injection time of 50 ms. A minimum ion intensity of 5000 was required to trigger an MS/MS spectrum. Dynamic exclusion was applied using a maximum exclusion list of 500 with one repeat count with a repeat duration of 30 s and exclusion duration of 15 s.

Raw MS files acquired from the mass spectrometer were processed using PEAKS software (Bioinformatics Solutions Inc.). Data was loaded into the software program and data from each fraction was refined to merge scans within 2 min and 10.0 ppm. Spectra with PEAKS filter scores <0.5 were excluded. De novo sequencing and database searching was done using a precursor mass cutoff of 10.0 ppm and a fragment mass tolerance of 0.6 Da. Carbidomethylation of cysteine (+57.02 Da) residues was selected as a fixed modification while variable modifications included 13C6-15N2 SILAC on K (8.01 Da), 13C6-15N4 SILAC on R (10.02), Oxidation of M (15.99). Label-free quantification was performed in PEAKS using SILAC labels.

Protein factors engaged in intense protein synthesis localize to the polysome (high ribosome density) fractions, while those present in the ribosome-free fraction are essentially disengaged from translation. As such, we used the ratio of protein abundance in the polysome to free fractions as our primary readout for translational engagement^43^. The ratio of polysomal to monosomal (40S/60S/80S) protein abundance was used as a secondary readout for translational intensity.

### MS Analysis for TMT-pSILAC

Three independent experiments for each RBP knockdown were pooled into a single sample and subjected to TMT-pSILAC MS analysis. MS sample preparation and runs were performed by the SPARC Biocentre, The Hospital for Sick Children (Toronto, Canada). Samples were reduced, alkylated, digested, and TMT labeled using the TMT10plex™ Isobaric Label Reagent Set (ThermoFisher Scientific, #90110) according to the manufacturer’s directions. Labeled peptides from all samples were combined and lyophilized. Peptides were then resuspended in 20 μl of ddH_2_O and subjected to high pH reversed-phase HPLC fractionation using a Waters XBridge C18 column. A 90 min gradient using buffer A (ddH_2_O, adjusted to pH 10 with ammonium hydroxide) and buffer B (80% acetonitrile, adjusted to pH 10 with ammonium hydroxide) was run as follows: 0–3 min 1–12% B; 3–60 min 12–30% B; 60–65 min 30–60% B; 65–70 min 60–99% B, 70–75 min 99–1% B, 75–90 min 1% B. Ultra violet (UV) absorbance was measured throughout the gradient at 214 nm and 280 nm using a Waters 2489 UV/Visible detector. Fractions were collected from the beginning of the gradient in 1.2 min intervals for 60 fractions.

For TMT-pSILAC of oxygen-sensitive RBPs, each of the 60 high pH fractions were lyophilized, resuspended in 0.1% trifluoroacetic acid, and loaded onto an Evotip C18 trap column (Evosep Biosystems, Denmark) as per manufacturer’s instructions. Samples were injected into an Orbitrap Fusion^TM^ Lumos^TM^ Tribrid^TM^ Mass Spectrometer (ThermoFisher Scientific) using an Evosep One LC instrument (Evosep Biosystems). The standard pre-set method of 60 samples per day was used. Peptides were introduced by nano-electrospray into the mass spectrometer. Data was acquired using the MultiNotch MS3 acquisition with synchronous precursor selection (SPS) with a cycle time of 2 s. MS1 acquisition was performed with a scan range of 550 *m/z*–1800 *m/z* with resolution set to 120,000, maximum injection time of 50 ms and AGC target set to 4e5. Isolation for MS2 scans was performed in the quadrupole, with an isolation window of 0.7. MS2 scans were done in the linear ion trap with a maximum injection time of 50 ms and a normalized collision energy of 35%. For MS3 scans, HCD was used, with a collision energy of 65% and scans were measured in the orbitrap with a resolution of 50,000, a scan range of 100 *m/z*–500 *m/z*, an AGC Target of 3e4, and a maximum injection time of 50 ms. Dynamic exclusion was applied using a maximum exclusion list of 500 with one repeat count with an exclusion duration of 45 s.

For TMT-pSILAC of LARP1, fractionated samples were concatenated from 60 samples to 15 samples by mixing early, middle, and late fractions together. Samples were analyzed on an Orbitrap Fusion^TM^ Lumos^TM^ Tribrid^TM^ Mass Spectrometer (ThermoFisher Scientific) outfitted with a nanospray and Evosep One LC system (Evosep). Lyophilized peptide mixtures were dissolved in 0.1% formic acid and loaded onto a C18 Evotip (Evosep). Samples were eluted and loaded onto a 15-C18 analytical column (100 μm ID, 3 μm beads) by Easy nLC1200 LC system (Thermo Scientific). A linear gradient of 0–42% Buffer A (0.1% Formic Acid in water) to Buffer B (80% acetonitrile, 0.1% formic acid) was used with a 90 min run time. Data was acquired using the MultiNotch MS3 acquisition with synchronous precursor selection (SPS) with a cycle time of 5 s. MS1 acquisition was performed with a scan range of 550 *m/z* –1800 *m/z* with resolution set to 120,000, maximum injection time of 50 ms and AGC target set to 4e5. Isolation for MS2 scans was performed in the quadrupole, with an isolation window of 0.6. MS2 scans were done in the linear ion trap with a maximum injection time of 50 ms and a normalized collision energy of 35%. For MS3 scans, HCD was used, with a collision energy of 65% and scans were measured in the orbitrap with a resolution of 50,000, a scan range of 100 *m/z*–500 *m/z*, an AGC Target of 3e4, and a maximum injection time of 50 ms. Dynamic exclusion was applied using a maximum exclusion list of 500 with one repeat count with an exclusion duration of 20 s.

For MS data analysis, raw files were processed using Proteome Discoverer 2.2 (Thermo Fisher Scientific). The MS data were searched against the Human Uniprot Database (downloaded April 10 2017) consisting of only reviewed entries using the Sequest HT and MS Amanda 2.0 search engines. For both search algorithms, the parent and fragment mass tolerances were set to 10 ppm and 0.6 Da, respectively. Methionine oxidation was considered as a variable modification, as was N-terminal acetylation at the protein terminus. Static modifications of TMT at the peptide N-terminus, and carbidomethylation of cysteines were also considered. When looking for all heavy labeled proteins, fixed modifications of Heavy TMT (237.177 Da) on Lysine and Heavy 13C(6)15N(4) label on arginine were set. For all identifications, TMT and Heavy TMT were considered as dynamic modifications on lysine residues, as were heavy arginine. In each case, 2 missed cleavages were allowed. A search was also performed with fixed modification of carbamidomethylation and variable modifications of TMT, oxidation and carbamylation to assess the extent of carbamylation. With 0.1% FDR at the peptide level, and including PTMs with a minimum ion intensity of >= 5%, 0.48% of TMT-tagged PSMs were identified with a carbamylation modification. Search engine results were also processed through Percolator with q-values set to 0.01 for strict and 0.05 for relaxed. TMT reporter ions were quantified using the Proteome Discoverer 2.2 reporter ions quantifier node with an integration tolerance of 20 ppm, on the MS order of MS3.

Translatome remodeling analysis was performed based on fold difference in protein abundance in each RBP-silenced sample compared to the average of all samples in a given condition (normoxia or hypoxia). A fold difference of at least 15% was considered biologically significant, based on targeted immunoblot validations of various proteins covering a broad range of TMT determined fold changes, and given that TMT-based measurements can be relatively compressed in range compared to other methods^62^.

### Immunoblot

SDS-PAGE was performed on Bolt^TM^ 4–12% Bis-Tris Plus pre-made gels (ThermoFisher Scientific) using the Mini Gel Tank system (ThermoFisher Scientific), and transferred to 0.2 μm Immuno-Blot^®^ PVDF membranes (Bio-Rad) using the Bolt^TM^ Mini Blot Module (ThermoFisher Scientific), all according to the manufacturer’s protocols. Chemiluminescent signals were developed using SuperSignal^TM^ West Pico PLUS chemiluminescent substrate (ThermoFisher Scientific), and captured on an Amersham Imager 600 (GE Healthcare Life Sciences). The auto capture function was used, whereby the machine performs a short pre-exposure to determine the optimal exposure time that yields the highest possible signal in the linear range of the camera below saturation. Densitometry was performed on non-saturated signals/images using ImageJ (NIH). Fold changes were normalized to β-actin loading controls. Antibodies: PCBP1 (Cell Signaling, #8534S; 1:1000 dilution); PCBP2 (Abnova, #H00005094; 1:1000 dilution); HuR (Cell Signaling, #12582S; 1:1000 dilution); hnRNP A2/B1 (2A2) (Proteintech, #14183-1-AP; 1:1000 dilution); PTBP1 (Cell Signaling, #8776 S; 1:1000 dilution); LARP1 (Bethyl Laboratories, #A302-087A; 1:1000 dilution); hnRNP A3 (Proteintech, #25142-1-AP; 1:1000 dilution); hnRNP C (Santa Cruz Biotechnology, #sc-32308; 1:1000 dilution); NDRG1 (Abcam, #ab37897; 1:2000 dilution); GLUT1 (Novus Biologicals, # NB110-39113; 1:1000 dilution); ALDOC (Proteintech, #14884-1-AP; 1:1000 dilution); F3 (Abcam, #ab104513; 1:1000 dilution); PAI-1 (Proteintech, #13801-1-AP; 1:1000 dilution); HIF-1α (Novus Biologicals, #NB100-105; 1:1000 dilution); HIF-2α (Novus Biologicals, #NB100-122; 1:1000 dilution); HIF-1β (Novus Biologicals, #NB100-110; 1:1000 dilution); puromycin (3RH11) (Kerafast, #EQ0001; 1:1000 dilution); eIF5B (Santa Cruz Biotechnology, #393564; 1:1000 dilution); β-actin (C4) (Santa Cruz Biotechnology, #sc-47778; 1:5000 dilution); Rabbit IgG isotype control (Novus Biologicals, #NB810-56910; 5 μg per immunoprecipitation reaction); Goat anti-rabbit IgG secondary, HRP-conjugated (Novus Biologicals, #NB7160; 1:10000 dilution for immunoblot); Goat anti-mouse IgG secondary, HRP-conjugated (Proteintech, #SA00001-1; 1:10000 dilution for immunoblot); Donkey anti-goat IgG secondary, HRP-conjugated (Novus Biologicals, #NB7357; 1:10000 dilution for immunoblot).

### RNA interference

Target-specific pools of four independent small interfering RNA (siRNA) species (siGENOME SMARTpool, Dharmacon) were transfected according to the manufacturer’s protocols at a final concentration of 50 nM using Effectene (Qiagen) for 48 h before treatments so as to ensure effective target knockdown prior to experimentation.

### Propidium iodide (PI) and fluorescein diacetate (FDA) staining

Live cells were incubated with 2.5 μg/ml each of PI and FDA for 30 min at 37 °C and washed twice with media before imaging by fluorescence microscopy (Keyence, BZ-X800). Fixed cells were incubated with 10 μg/ml Hoechst 33342 (ThermoFisher Scientific) for 30 min at 37 °C and washed twice with PBS (−/−) before imaging by fluorescence microscopy. Cell were counted based on the number of Hoechst-stained nuclei per field.

### Subcellular fractionation

Subcellular fractionation was performed according to the published protocol of using 0.1% NP-40 (Calbiochem) to separate cytosolic and nuclear fractions^117^. Briefly, cells were washed twice with ice-cold PBS in the presence of protease inhibitors (ThermoFisher Scientific, #78430), and then lysed with 0.1% NP-40. After trituration, an aliquot of the lysate was removed (whole-cell lysate) immediately, mixed with 4X Laemmli buffer, and stored on ice. Following centrifugation (pulse-spin for 10 s) of the remaining cell lysate, an aliquot of the supernatant was removed (cytoplasmic fraction), mixed with 4X Laemmli buffer, boiled, and stored at −80 °C. Nuclei pellet was resuspended with 0.1% NP-40, centrifuged to remove supernatant, and resuspended in 4X Laemmli buffer. Nuclei pellet and whole-cell lysate were then sonicated (20 kHz, 2 pulses, 8 s each), and stored at −80 °C.

### Glucose uptake, lactate production, and cell viability

Measurements of glucose uptake and lactate production were performed in 96-well cell culture plates using the Glucose Uptake-Glo^TM^ (Promega) and Lactate-Glo^TM^ (Promega) assays, respectively, according to the manufacturer’s protocols. These assays were multiplexed with cell viability measurements from the same starting cell lysates using the CellTiter-Glo 2.0 assay (Promega), according to the manufacturer’s protocols.

### Gene ontology (GO) enrichment analysis

GO enrichment analysis was performed using the online Database for Annotation, Visualization and Integrated Discovery (DAVID) bioinformatics resource (v6.8)^118^.

### Co-immunoprecipitation

HA-HIF-1α and HA-HIF-2α plasmids (in pcDNA3 backbone) were gifts from William Kaelin (Addgene plasmid # 18949 and # 18950, respectively)^119^. Plasmids were transfected for 24 h using Effectene (Qiagen) before treatment and co-immunoprecipitation (co-IP). HA-HIF-1α and HA-HIF-2α co-IPs were performed using the Pierce™ HA-Tag Magnetic IP/Co-IP Kit (ThermoFisher Scientific) according to the manufacturer’s recommendations. HuR co-IPs were performed using the Pierce™ Classic Magnetic IP/Co-IP Kit (ThermoFisher Scientific) according to the manufacturer’s recommendations. 5 μg of HuR-specific antibody (Cell Signaling, #12582S) or rabbit IgG isotype control (Novus Biologicals, #NB810-56910) and 1 mg of total protein lysate were used for each reaction.

### RNA sequencing analysis

Normoxic and hypoxic translatome RNA sequencing analysis was performed by Exiqon (Denmark). Equal volumes of relevant ribosome density fractionated fractions were combined to yield the free, 40/60/80S, and polysome samples, respectively. Library preparation was done using TruSeq® Stranded mRNA Sample preparation kit (Illumina inc). The starting material (100 ng) of total RNA was mRNA enriched using the oligo-dT bead system. The isolated mRNA was subsequently fragmented using enzymatic fragmentation. Then first-strand synthesis and second-strand synthesis were performed and the double-stranded cDNA was purified (AMPure XP, Beckman Coulter). The cDNA was end repaired, 3′ adenylated and Illumina sequencing adapters ligated onto the fragments ends, and the library was purified (AMPure XP). The mRNA stranded libraries were pre-amplified with PCR and purified (AMPure XP). The libraries size distribution was validated and quality inspected on a Bioanalyzer high sensitivity DNA chip (Agilent Technologies). High quality libraries were quantified using qPCR, the concentration normalized and the samples pooled according to the project specification (number of reads). The library pool(s) were re-quantified with qPCR and optimal concentration of the library pool used to generate the clusters on the surface of a flow cell before sequencing (single-end, 50 bp runs at a depth of ≥30 million reads) on Nextseq500/High Output sequencing kit (51 cycles according to the manufacturer instructions (Illumina Inc).

For HuR, HIF-1α, and HIF-2α silencing experiments: equal volumes of relevant ribosome density fractionated fractions were combined to yield the free, 40/60/80S, and polysome samples, respectively. Poly(A) RNA selection, library preparation, and RNA sequencing were performed by the Sylvester Comprehensive Cancer Center Oncogenomics Core Facility, using the KAPA Stranded mRNA-Seq Kit (KAPA Biosystems) and NextSeq 500 High Output Kit v2 (Illumina). Paired-end (2 × 75 bp) sequencing runs at a depth of ≥50 million reads were performed on the libraries using the NextSeq 500 system (Illumina). Reads were quality-checked using the Cutadapt software^120^. Passed reads were aligned to the human genome (GRCh38 assembly) using the Spliced Transcripts Alignment to a Reference (STAR) software with default parameters^121^. Cufflinks analysis was performed to determine FPKM values for reference annotated genes^122^.

To analyze the effects of RBPs, TE was defined as the ratio of FPKM in polysome fractions to that in free/monosome fractions. Steady-state RNA level was defined as the aggregate FPKM across all fractions. ΔTE and ΔRNA were defined as the ratio of TE and RNA levels, respectively, in RBP-silenced sample to non-silencing (NS) control sample. A two-fold difference was considered biologically significant for RBP-mediated effects.

### qRT-PCR

First-strand cDNA synthesis was performed using the High-Capacity cDNA Reverse Transcription Kit (Thermo Fisher Scientific), according to the manufacturer’s protocols. qRT-PCR was performed using the PowerUp^TM^ SYBR^®^ Green Master Mix (Thermo Fisher Scientific) and a StepOnePlus^TM^ Real-Time PCR System (Thermo Fisher Scientific). Relative changes in expression were calculated using the comparative Ct (ΔΔCt) method. 18S rRNA was measured as an internal control for changes in RNA levels. Primer sequences are available upon request.

### Primer sequences

Human targets: GLUT1 (Forward 5′-TGGCCGTGGGAGGAGCAGTG-3′; Reverse 5′-GCGGTGGACCCATGTCTGGTTG-3′), HK2 (Forward 5′-CCCCTGCCACCAGACTAAAC-3′; Reverse 5′-CAAAGTCCCCTCTCCTCTGG-3′), GPI (Forward 5′-CGCCCAACCAACTCTATTGTG-3′; Reverse 5′-TCCACTCCCCACTGGTCAAAG-3′), PFKP (Forward 5′-ACCAACCTGTGTGTGATCGG-3′; Reverse 5′-TATCGATCTGGCCGTTCCTG-3′), ALDOA (Forward 5′-GGTGCAGCTCCCGGAC-3′; Reverse 5′-TGAAGACGATGGCAGGGATG-3′), ALDOC (Forward 5′-TCACGTAGCTCTGCGACATC-3′; Reverse 5′-CATGGTGACAGCTCCCTGTG-3′), GAPDH (Forward 5′-CTGCACCACCAACTGCTT-3′; Reverse 5′-GTCTTCTGGGTGGCAGTG-3′), PGK (Forward 5′-GTGCCCATGCCTGACAAGTAC-3′; Reverse 5′-TGGGCCTACACAGTCCTTCAAG-3′), PGAM1 (Forward 5′-CCGGGGCATTGTCAAGCATCT-3′; Reverse 5′-AGTCGGCAGGTTCAGCTCCATGA-3′), ENO1 (Forward 5′-TGGCTGGCAACTCTGAAGTCATC-3′; Reverse 5′-TGCACCGACTGGGAGGATCAT-3′), ENO2 (Forward 5′-CATGCGACTAGGTGCAGAGG-3′; Reverse 5′-AGCTCCAAGGCTTCACTGTT-3′), PKM (Forward 5′-CTGTGGCTGGACTACAAGAAC-3′; Reverse 5′-CCTCCGTCACCAGGAAGTC-3′), NDRG1 (5′-GCAGGCGCCTACATCCTAACT-3′); Reverse 5′-GCTTGGGTCCATCCTGAGATCTT-3′); VEGFA (Forward 5′-GCAGACCAAAGAAAGATAGACCAAG-3′; Reverse 5′-CGCCTCGGCTTGTCACAT-3′), 18S (Forward 5′-AGGAATTGACGGAAGGGCAC-3′; Reverse 5′-GGACATCTAAGGGCATCACA-3′).

*C. elegans* targets: fgt-1 (Forward 5′-CTGCAGGAGGTCCAGTCAAG-3′; Reverse 5′-TGATAAGCCCTCCTGGAGCA-3′), hxk-2 (Forward 5′-TATGAAGCTGGCGGTCACTG-3′; Reverse 5′-TAATCCTCCGTAGCGTTGGC-3′), gpi-1 (Forward 5′-TGCTCGGCTACCTTTCTGAC-3′; Reverse 5′-CAGAGACATGGCTTTTTCGGTG-3′), pfk-1.1 (Forward 5′-AAGTCAGCCCATACTGCGAC-3′; Reverse 5′-GCCATCCACCATTGCTCTGA-3′), aldo-1 (Forward 5′-TGAGCCAGAGATCCTCCCAG-3′; Reverse 5′-CCCTCGAGGAAGACGTGATG-3′), gpd-1 (Forward 5′-AAGAAGGTAGTCAAGGCCGC-3′; Reverse 5′-TTGTCGTACCAAGAGACGAGC-3′), enol-1 (Forward 5′-TCTGTCTGGACAAAGGATGCC-3′; Reverse 5′-AAGCTCCAGATGGAACTGCG-3′). VC3835 genotyping primers (Forward 5′-CTCGGAAGATGGGGGGGAAG-3′; Reverse 5′-GGAGCAAGGTGAGATGAC-3′).

### Global protein synthesis measurement

Global protein synthesis was measured by puromycin (ThermoFisher Scientific) incorporation (1 μg/ml final concentration for 20 min), followed by protein extraction using RIPA buffer (ThermoFisher Scientific) and immunoblot analysis with an anti-puromycin antibody (3RH11) (Kerafast, #EQ0001).

### Immunocytochemistry

Cells grown on glass coverslips were fixed with 4% formaldehyde (in PBS) for 10 min, washed with PBS, and solubilized with 0.5% triton (in PBS) for 10 min. Cells were blocked for 30 min in 5% FBS (in PBS), and incubated for 1 hr at 37 °C in primary antibody (Novus Biologicals, #NB100-122 (HIF-2α), #AF1935 (HIF-1α); 1:100 in 5% FBS). Cells were then washed and incubated for 1 h at 37 °C in secondary antibody (ThermoFisher, #A-11008, #A-11055; 1:500 dilution in 5% FBS). Cells were washed 3× for 10 min each with PBS. DAPI stain (1/10,000) was added during the second wash. Coverslips were mounted with Fluoromount (Millipore Sigma) and images were taken on a fluorescent microscope (Keyence BZ-X800).

### Statistical analysis

All experiments were performed at least three independent times, unless otherwise stated. Quantitation of microscopy-based data was performed on at least 10 representative images. Statistical analyses performed are indicated in relevant figure legends. Statistical significance was defined as *p* < 0.05. For Gene Ontology analyses (performed using the DAVID online bioinformatic resource^118^), false discovery rate was controlled by the Benjamini–Hochberg procedure to produce *p* values adjusted for multiple comparisons.

### Reporting summary

Further information on research design is available in the Nature Research Reporting Summary linked to this article.

## Supplementary information

Supplementary Information

Reporting Summary

## Data Availability

The data that support this study are available from the corresponding author upon reasonable request. Mass spectrometry datasets are available via the ProteomeXchange accessions: PXD011979, PXD006799. MS data were searched against the Human Uniprot Database (https://www.uniprot.org). RNA sequencing datasets are available via the NCBI GEO accessions: GSE128541, GSE128547, GSE128555. The source data underlying Figs. 1c–h, 2b–e, 2g, 3e, 3f, 4a, 4d, 5a, 5f–h, 6c–g, and Supplementary Figs. 1c, 1e, 1g, 1i, 2b, 2e–h, 3c, 3g, 4d, 4e, 4g, 5l, 5m, 6j, 6k, 7d are provided as a Source data file.

## Source data

Source Data
