# Supplementary material for: A network of RNA-binding proteins controls translation efficiency to activate anaerobic metabolism
Source: Nat Commun. 2020 May 29;11:2677. doi: 10.1038/s41467-020-16504-1 (PMC7260222; doi:10.1038/s41467-020-16504-1)
Supplement: Supplementary file 1 — Supplementary Information [file 41467_2020_16504_MOESM1_ESM.pdf]

Supplementary Information

A Network of RNA-Binding Proteins Controls Translation Efficiency to Activate Anaerobic Metabolism

Ho *et al.*

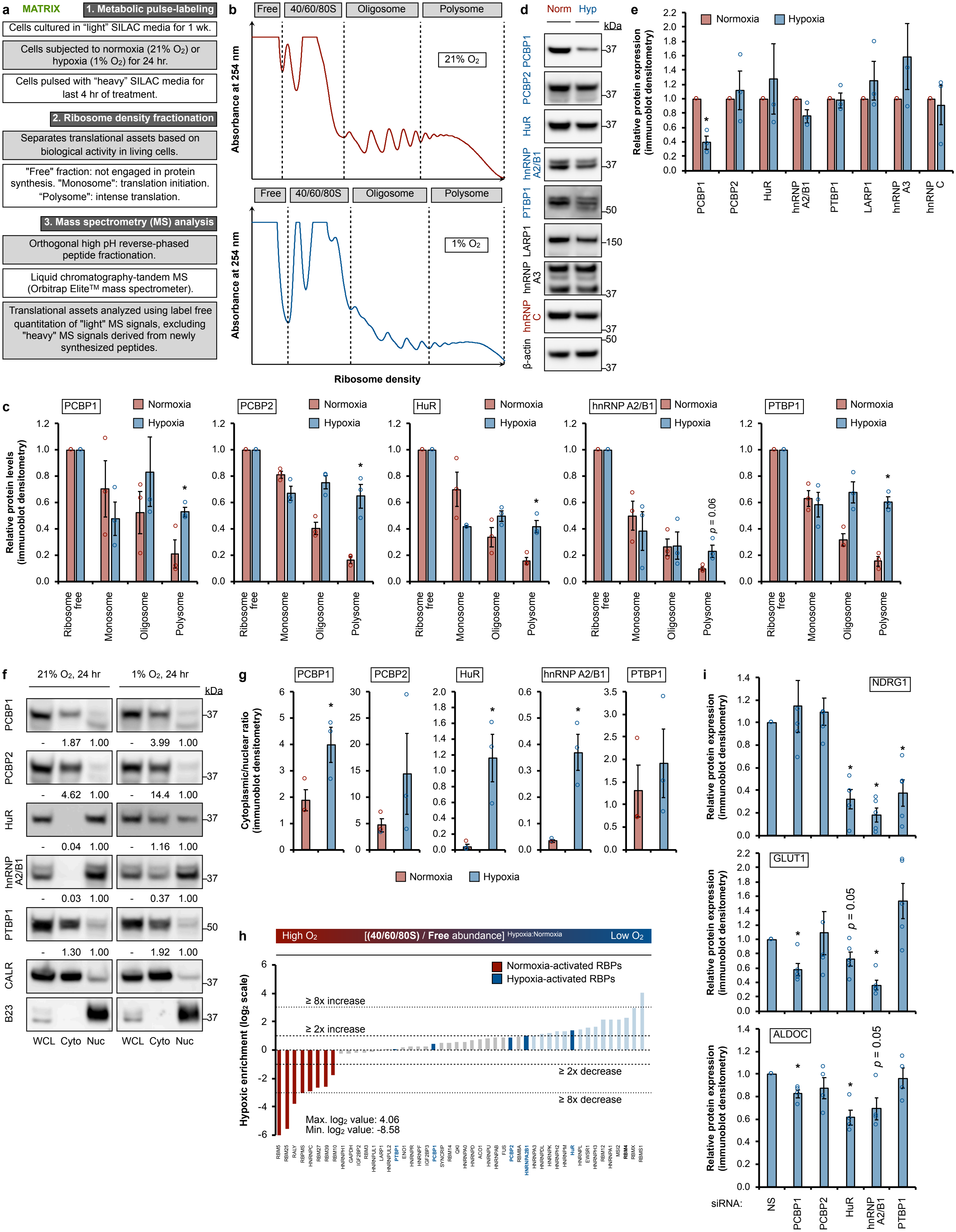

a MATRIX
1. Metabolic pulse-labeling
Cells cultured in "light" SILAC media for 1 wk.
Cells subjected to normoxia (21% O₂) or hypoxia (1% O₂) for 24 hr.
Cells pulsed with "heavy" SILAC media for last 4 hr of treatment.
2. Ribosome density fractionation
Separates translational assets based on biological activity in living cells.
"Free" fraction: not engaged in protein synthesis. "Monosome": translation initiation. "Polysome": intense translation.
3. Mass spectrometry (MS) analysis
Orthogonal high pH reverse-phased peptide fractionation.
Liquid chromatography-tandem MS (Orbitrap Elite™ mass spectrometer).
Translational assets analyzed using label free quantitation of "light" MS signals, excluding "heavy" MS signals derived from newly synthesized peptides.
b
Free
40/60/80S
Oligosome
Polysome
21% O₂
1% O₂
Absorbance at 254 nm
Ribosome density
d
Norm
Hyp
kDa
PCBP1
PCBP2
HuR
hnRNP A2/B1
PTBP1
LARP1
hnRNP A3
hnRNP C
β-actin
e
Normoxia
Hypoxia
Relative protein expression (immunoblot densitometry)
PCBP1
PCBP2
HuR
hnRNP A2/B1
PTBP1
LARP1
hnRNP A3
hnRNP C
c
PCBP1
PCBP2
HuR
hnRNP A2/B1
PTBP1
Relative protein levels (immunoblot densitometry)
Ribosome free
Monosome
Oligosome
Polysome
p = 0.06
f
21% O₂, 24 hr
1% O₂, 24 hr
WCL Cyto Nuc
- 1.87 1.00 - 3.99 1.00
- 4.62 1.00 - 14.4 1.00
- 0.04 1.00 - 1.16 1.00
- 0.03 1.00 - 0.37 1.00
- 1.30 1.00 - 1.92 1.00
CALR
B23
g
Cytoplasmic/nuclear ratio (immunoblot densitometry)
Normoxia
Hypoxia
h
High O₂
[(40/60/80S) / Free abundance] Hypoxia:Normoxia
Low O₂
Normoxia-activated RBPs
Hypoxia-activated RBPs
≥ 8x increase
≥ 2x increase
≥ 2x decrease
≥ 8x decrease
Max. log₂ value: 4.06
Min. log₂ value: -8.58
Hypoxic enrichment (log₂ scale)
i
NDRG1
GLUT1
ALDOC
Relative protein expression (immunoblot densitometry)
p = 0.05
siRNA:
NS
PCBP1
PCBP2
HuR
hnRNP A2/B1
PTBP1

Supplementary Figure 1. (**a**) Schematic of MATRIX (mass spectrometry analysis of active translation factors using ribosome density fractionation and isotopic labeling experiments) workflow. (**b**) Ribosome density profiles of normoxic (red) and hypoxic (blue) U87MG. (**c**) Densitometry of immunoblots in Fig. 1e. Asterisk denotes statistical significance calculated using two-sided Student's t-tests compared to normoxia polysome fraction. Exact p values: PCBP1 ($p = 0.045$), PCBP2 ($p = 0.035$), HuR ($p = 0.046$), PTBP1 ($p = 0.0008$). Data represent mean±SEM (error bars) of three independent experiments (n=3). (**d**) Representative immunoblots and (**e**) densitometry of U87MG whole cell lysates. N: normoxia (21% O_2); H: hypoxia (1% O_2, 24 hr). Asterisk denotes statistical significance calculated using two-sided Student's t-tests compared to corresponding normoxia control. Exact p value: PCBP1 ($p = 0.02$). Data represent mean±SEM of three independent experiments (error bars) (n=3). (**f**) Representative immunoblots and (**g**) densitometry of U87MG subcellular fractionated samples. WCL: whole cell lysate; Cyto: cytoplasmic; Nuc: nuclear. Asterisk denotes statistical significance calculated using two-sided Student's t-tests compared to corresponding normoxia control. Exact p values: PCBP1 ($p = 0.04$), HuR ($p = 0.038$), hnRNP A2/B1 ($p = 0.017$). Data represent mean±SEM (error bars) of three independent experiments (n=3). (**h**) MATRIX readout of relative RBP engagement in translation initiation (ratio of (40/60/80S)/free protein abundance) in hypoxic (1% O_2, 24 hr, blue) versus normoxic (21% O_2, 24 hr, red) U87MG. (**i**) Densitometry of immunoblots in Fig. 1f. Asterisk denotes statistical significance calculated using two-sided Student's t-tests compared to NS control. Exact p values: NDRG1: HuR siRNA ($p = 0.001$), hnRNP A2/B1 siRNA ($p = 0.0001$), PTBP1 siRNA ($p = 0.006$); GLUT1: PCBP1 siRNA ($p = 0.008$), hnRNP A2/B1 siRNA ($p = 0.0007$); ALDOC: PCBP1 siRNA ($p = 0.017$), HuR siRNA ($p = 0.01$). Data represent mean±SEM of four independent experiments (n=4). Source data are provided as a Source Data file.

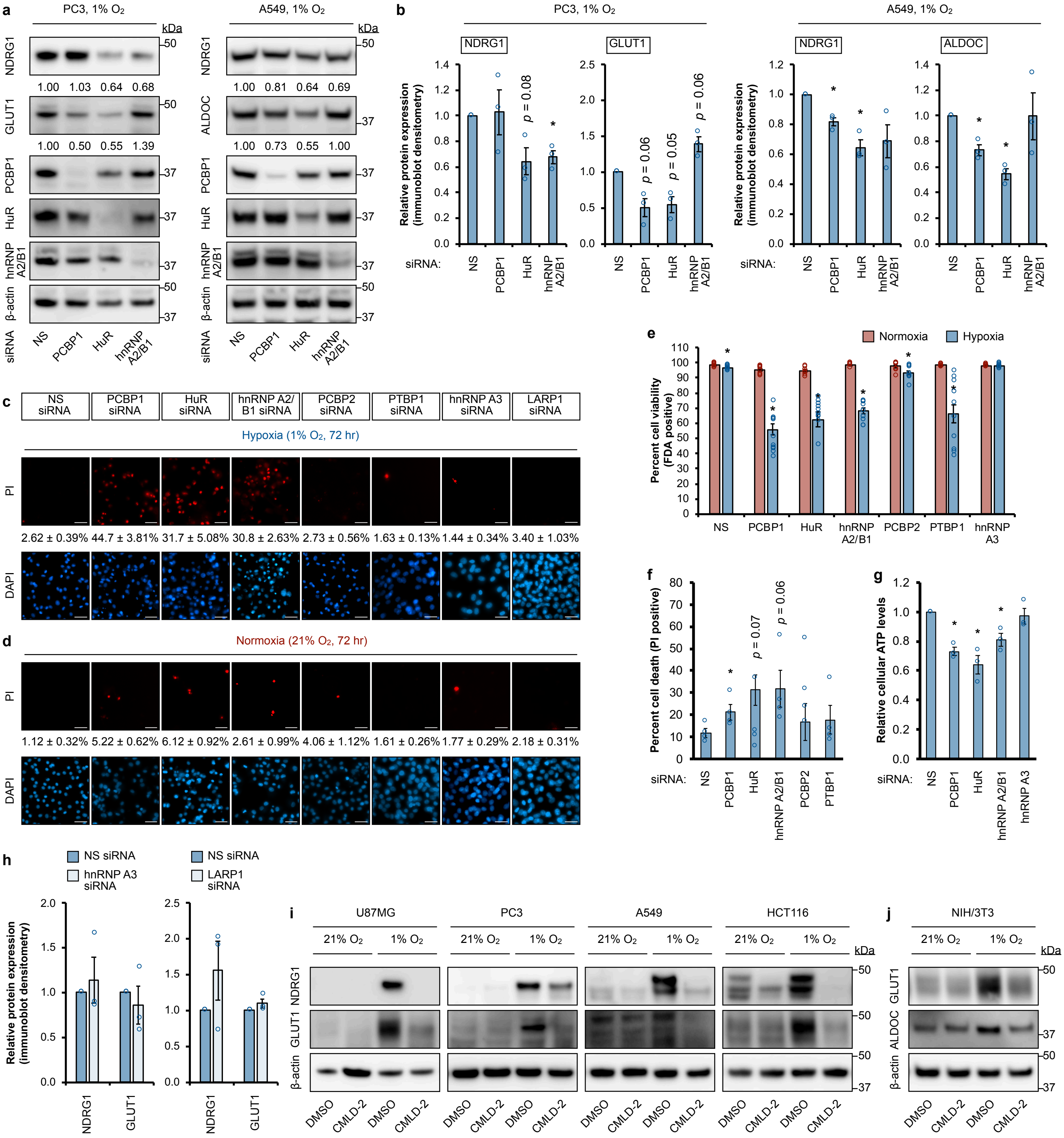

a
PC3, 1% O2
A549, 1% O2
kDa
NDRG1
1.00 1.03 0.64 0.68
1.00 0.81 0.64 0.69
GLUT1
ALDOC
1.00 0.50 0.55 1.39
1.00 0.73 0.55 1.00
PCBP1
HuR
hnRNP A2/B1
β-actin
siRNA
NS
PCBP1
HuR
hnRNP A2/B1
b
PC3, 1% O2
A549, 1% O2
NDRG1
GLUT1
NDRG1
ALDOC
Relative protein expression (immunoblot densitometry)
p = 0.08
p = 0.06
p = 0.05
p = 0.06
siRNA:
c
NS siRNA
PCBP1 siRNA
HuR siRNA
hnRNP A2/B1 siRNA
PCBP2 siRNA
PTBP1 siRNA
hnRNP A3 siRNA
LARP1 siRNA
Hypoxia (1% O2, 72 hr)
PI
2.62 ± 0.39% 44.7 ± 3.81% 31.7 ± 5.08% 30.8 ± 2.63% 2.73 ± 0.56% 1.63 ± 0.13% 1.44 ± 0.34% 3.40 ± 1.03%
DAPI
d
Normoxia (21% O2, 72 hr)
PI
1.12 ± 0.32% 5.22 ± 0.62% 6.12 ± 0.92% 2.61 ± 0.99% 4.06 ± 1.12% 1.61 ± 0.26% 1.77 ± 0.29% 2.18 ± 0.31%
DAPI
e
Normoxia
Hypoxia
Percent cell viability (FDA positive)
NS
PCBP1
HuR
hnRNP A2/B1
PCBP2
PTBP1
hnRNP A3
f
Percent cell death (PI positive)
p = 0.07
p = 0.06
siRNA:
NS
PCBP1
HuR
hnRNP A2/B1
PCBP2
PTBP1
g
Relative cellular ATP levels
siRNA:
NS
PCBP1
HuR
hnRNP A2/B1
hnRNP A3
h
NS siRNA
hnRNP A3 siRNA
NS siRNA
LARP1 siRNA
Relative protein expression (immunoblot densitometry)
NDRG1
GLUT1
i
U87MG
PC3
A549
HCT116
21% O2
1% O2
kDa
NDRG1
GLUT1
β-actin
DMSO
CMLD-2
j
NIH/3T3
21% O2
1% O2
kDa
GLUT1
ALDOC
β-actin
DMSO
CMLD-2

Supplementary Figure 2. (**a**) Representative immunoblots and (**b**) densitometry of PC3 and A549 treated with indicated siRNAs. NS: Non-silencing. Asterisk denotes statistical significance calculated using two-sided Student's t-tests compared to NS control. Exact p values: NDRG1 (PC3): hnRNP A2/B1 siRNA ($p = 0.024$); NDRG1 (A549): PCBP1 siRNA ($p = 0.021$), HuR siRNA ($p = 0.022$); ALDOC (A549): PCBP1 siRNA ($p = 0.026$), HuR siRNA ($p = 0.009$). Data represent mean±SEM (error bars) of three independent experiments (n=3). Representative images of (**c**) hypoxic versus (**d**) normoxic U87MG treated with indicated siRNAs. Scale bar: 50 µm. Quantitation represents mean±SEM (error bars) (n=10 fields over three independent experiments). (**e**) Cell viability measurements by FDA staining in U87MG treated with indicated siRNAs. Hypoxia: 1% O_2, 72 hr. Asterisk denotes statistical significance calculated using two-sided Student's t-tests compared to corresponding normoxia control. Exact p values: NS siRNA ($p = 0.004$), PCBP1 siRNA ($p = 6.74e-07$), HuR siRNA ($p = 0.0002$), hnRNP A2/B1 siRNA ($p = 5.17e-08$), PCBP2 siRNA ($p = 0.026$), PTBP1 siRNA ($p = 0.0005$), hnRNP A3 siRNA ($p = 0.551$). Data represent mean±SEM (error bars) (n=10 fields over three independent experiments). (**f**) Cell death measurements by propidium iodide (PI) staining in U87MG treated with indicated siRNAs. Hypoxia: 1% O_2, 24 hr. Asterisk denotes statistical significance calculated using two-sided Student's t-tests compared to NS control. Exact p value: PCBP1 siRNA ($p = 0.029$). Data represent mean±SEM (error bars) of four independent experiments (n=4). (**g**) Cell viability measurements based on cellular ATP levels using the CellTiter-Glo® 2.0 Assay (Promega) in U87MG treated with indicated siRNAs. Hypoxia: 1% O_2, 24 hr. Asterisk denotes statistical significance calculated using two-sided Student's t-tests compared to NS control. Exact p values: PCBP1 siRNA ($p = 0.01$), HuR siRNA ($p = 0.031$), hnRNP A2/B1 siRNA ($p = 0.047$). Data represent mean±SEM (error bars) of three independent experiments (n=3). (**h**) Densitometry of immunoblots in Fig. 1h. Data represent mean±SEM (error bars) of three independent experiments (n=3). Representative immunoblots of (**i**) human cell lines and (**j**) NIH/3T3 mouse embryonic fibroblasts treated with HuR-inhibiting compound CMLD-2 or DMSO vehicle control. Three independent experiments (n=3) were performed with similar results. Source data are provided as a Source Data file.

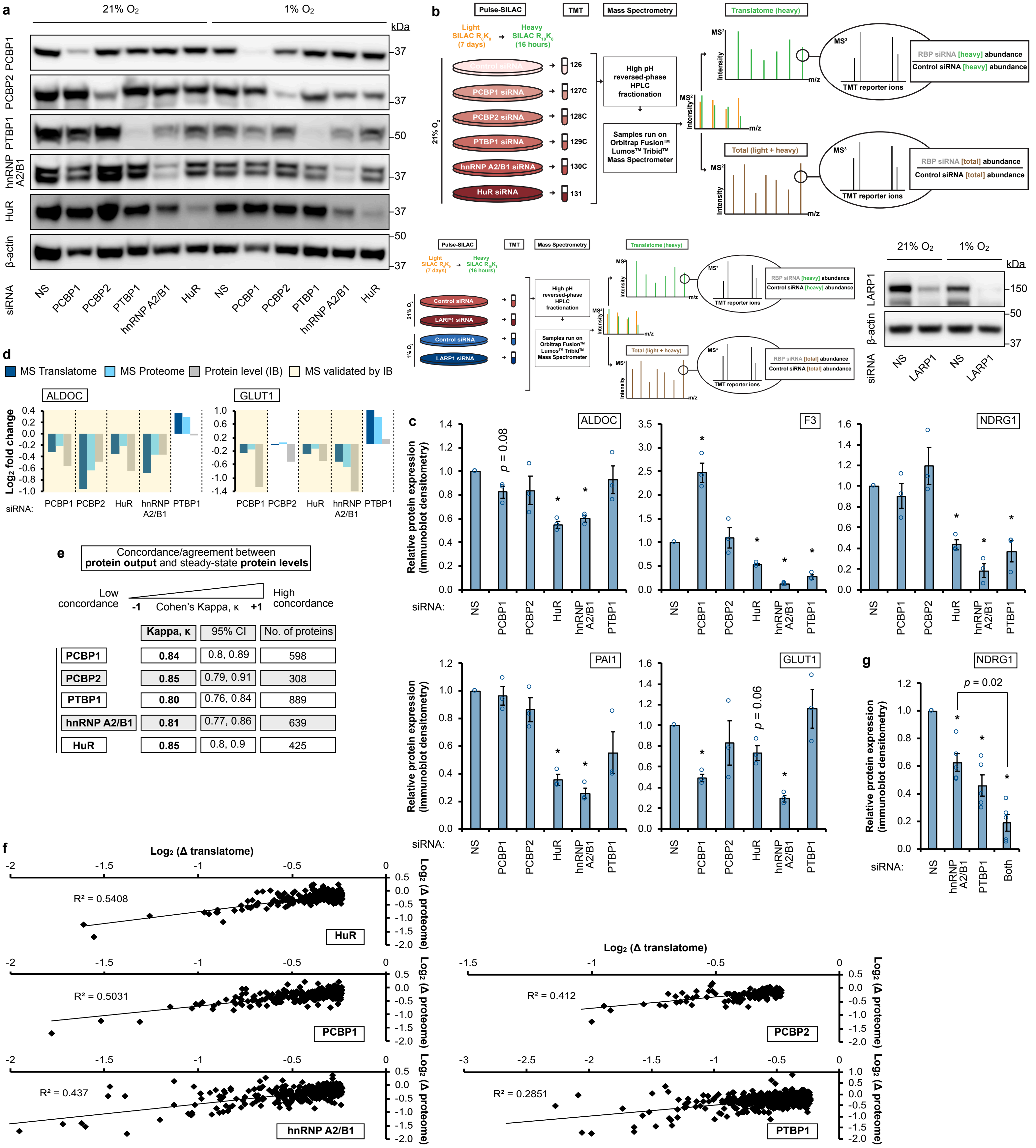

	Kappa, κ	95% CI	No. of proteins
PCBP1	0.84	0.8, 0.89	598
PCBP2	0.85	0.79, 0.91	308
PTBP1	0.80	0.76, 0.84	889
hnRNP A2/B1	0.81	0.77, 0.86	639
HuR	0.85	0.8, 0.9	425

Supplementary Figure 3. (**a**) Representative control immunoblots of U87MG for RBP-dependent TMT-pSILAC translatome analysis. NS: Non-silencing. Three independent experiments (n=3) were performed with similar results. (**b**) Schematic of translatome analysis strategy using TMT-pSILAC in normoxic U87MG (upper panel) and LARP1 TMT-pSILAC in hypoxic and normoxic U87MG (lower panels). (**c**) Densitometry of immunoblots in Fig. 2e. Asterisk denotes statistical significance calculated using two-sided Student's t-tests compared to NS control. Exact p values: ALDOC: HuR siRNA (p = 0.0044), hnRNP A2/B1 siRNA (p = 0.0045); FS: PCBP1 siRNA (p = 0.019), HuR siRNA (p = 0.0021), hnRNP A2/B1 siRNA (p = 0.0002), PTBP1 siRNA (p = 0.0033); NDRG1: HuR siRNA (p = 0.0066), hnRNP A2/B1 siRNA (p = 0.0072), PTBP1 siRNA (p = 0.025); PAI1: HuR siRNA (p = 0.0033), hnRNP A2/B1 siRNA p = 0.0029); GLUT1: PCBP1 siRNA (p = 0.0053), hnRNP A2/B1 siRNA (p = 0.002). Data represent mean±SEM (error bars) of three independent experiments (n=3). (**d**) Agreement between translatome and proteome readouts by MS and immunoblot analyses of representative targets. Three independent experiments were pooled into a single sample for MS measurement. (**e**) Concordance analysis using Cohen's Kappa statistic between RBP-dependent changes in protein output and steady-state protein levels under hypoxic conditions. (**f**) Coefficient of determination (R^2) analysis of each RBP-dependent change in protein output versus steady-state protein levels as measured by TMT-pSILAC. (**g**) Densitometry of immunoblots in Fig. 2g. Asterisk denotes statistical significance calculated using two-sided Student's t-tests compared to NS control. Exact p values: hnRNP A2/B1 siRNA (p = 0.0042), PTBP1 siRNA (p = 0.0019), both siRNA (p = 0.00018). Data represent mean±SEM (error bars) of five independent experiments (n=5). Source data are provided as a Source Data file.

Canonical hypoxia/HIF-inducible targets regulated by RBPs

Gene	PCBP1	PCBP2	HuR	hnRNP A2/B1	PTBP1
ALDOA	✓	-	-	-	-
ALDOC	✓	✓	✓	✓	-
ANXA2	✓	✓	-	-	-
BNIP3	-	✓	✓	✓	-
CA12	-	-	✓	✓	-
CA9	-	-	-	✓	-
CTSA	✓	-	✓	-	-
EGLN1	-	-	✓	-	-
ENO1	✓	-	-	-	-
ENO2	-	✓	-	✓	-
F3	-	-	✓	✓	✓
FAM162A	✓	✓	✓	✓	-
GAPDH	✓	-	-	✓	-
GBE1	-	-	-	✓	-
GPI	✓	✓	-	-	-
GYS1	-	-	-	✓	-
HK2	-	-	-	✓	-
HMOX1	-	-	✓	-	-
IGFBP3	-	-	-	✓	-
ITGA5	-	-	-	✓	-
LGALS3	✓	-	✓	✓	✓
LOX	-	-	✓	✓	-
LRP1	-	-	✓	✓	-
MIF	✓	-	✓	-	-
NAMPT	-	-	-	✓	✓
NDRG1	-	-	-	✓	✓
NFKB1	-	✓	-	✓	✓
P4HA1	-	✓	-	-	-
P4HA2	✓	✓	✓	-	-
P4HB	-	-	✓	-	-
PFKP	-	-	-	✓	-
PGAM1	✓	✓	-	-	-
PGK1	✓	-	-	-	-
PKM	-	✓	-	-	-
PLAU	✓	-	✓	-	-
PLOD2	-	-	✓	-	-
RUVBL2	-	-	-	-	✓
SERPINE1	-	-	-	✓	✓
SLC16A3	-	-	-	✓	-
SLC2A1	✓	-	✓	✓	-
SLC2A3	-	-	✓	✓	✓
TFRC	-	✓	-	-	-
TPI1	-	-	-	✓	-
VDAC1	-	-	✓	-	-

Canonical hypoxia/HIF-inducible targets not regulated by RBPs

Gene	PCBP1	PCBP2	HuR	hnRNP A2/B1	PTBP1
DNAJB6	-	-	-	-	-
EIF4EBP1	-	-	-	-	-
HK1	-	-	-	-	-
LDHA	-	-	-	-	-
PFKFB4	-	-	-	-	-
PFKL	-	-	-	-	-
VEGFA	-	-	-	-	-

Supplementary Table 1. Canonical hypoxia/HIF-inducible proteins regulated (left) and not regulated (right) by hypoxia-adaptive RBPs, determined by TMT-pSILAC.

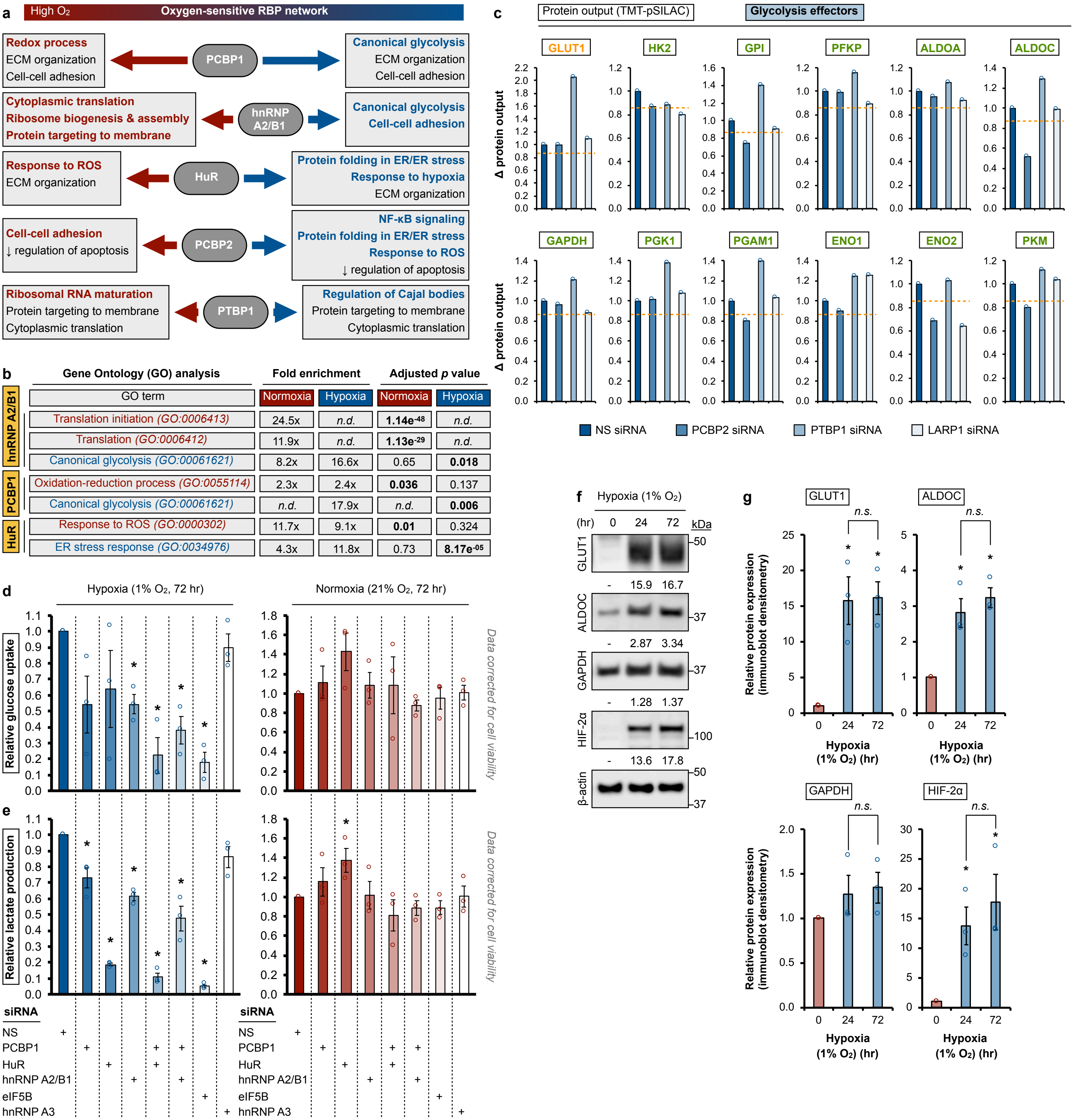

Supplementary Figure 4. (**a**) Oxygen-dependent remodeling of RBP-regulated pathways based on Gene Ontology (GO) pathway enrichment analysis. (**b**) GO analysis of representative enriched biological processes for down-regulated proteins when indicated RBPs are silenced. (**c**) Protein output regulation of each glycolytic effector by PCBP2, PTBP1, and LARP1, as determined by TMT-pSILAC (three independent experiments pooled into a single sample for measurement). Orange dotted line represents the validated regulatory threshold. Measurements of (**d**) glucose uptake and (**e**) lactate production in U87MG treated with indicated siRNAs. NS: Non-silencing. Asterisk denotes statistical significance calculated using two-sided Student's t-tests compared to NS control. Exact p values (d): hnRNP A2/B1 siRNA (p = 0.0088), PCBP1 + HuR siRNA (p = 0.0096), PCBP1 + hnRNP A2/B1 siRNA (p = 0.0093), eIF5B siRNA (p = 0.0027). Exact p values (e): Hypoxia: PCBP1 siRNA (p = 0.024), HuR siRNA (p = 5.39e-05), hnRNP A2/B1 siRNA (p = 0.0022), PCBP1 + HuR siRNA (p = 0.00048), PCBP1 + hnRNP A2/B1 siRNA (p = 0.011), eIF5B siRNA (p = 5.03e-05); Normoxia: HuR siRNA (p = 0.047). Data represent mean±SEM (error bars) of three independent experiments (n=3). (**f**) Representative immunoblots and (**g**) densitometry of U87MG whole cell lysates. Asterisk denotes statistical significance calculated using two-sided Student's t-tests compared to normoxia. Exact p values: GLUT1: 24 hr (p = 0.023), 72 hr (p = 0.011); ALDOC: 24 hr (p = 0.023), 72 hr (p = 0.0076); HIF-2α: 24 hr (p = 0.029), 72 hr (p = 0.034). Data represent mean±SEM (error bars) of three independent experiments (n=3). Source data are provided as a Source Data file.

RBP	Glycolysis enzyme/ glucose transporter	CLIP-seq evidence	Transcript-RBP binding region(s)	Publication(s) (PMID or GEO accession)
hnRNP A2/B1	Glucose transporter 1	Yes	*3'-UTR*	*Neuron* 92:780; 2016 (27773581)
	Hexokinase 1/2	Yes	*3'-UTR*	*Neuron* 92:780; 2016 (27773581)
	Phosphofructokinase	Yes	*3'-UTR*	*Neuron* 92:780; 2016 (27773581)
	Aldolase A/C	Yes	*3'-UTR, 5'UTR, coding region*	*Neuron* 92:780; 2016 (27773581)
	Glyceraldehyde 3-phosphate dehydrogenase	Yes	*3'-UTR, 5'UTR, coding region*	*Neuron* 92:780; 2016 (27773581)
	Enolase 2	Yes	*3'-UTR, 5'UTR, coding region*	*Neuron* 92:780; 2016 (27773581)
PCBP2	Glucose-6-phosphate isomerase	Yes	*3'-UTR*	*Nature* 489:57; 2012 (22955616)
	Aldolase C	Yes	*3'-UTR*	*Nature* 489:57; 2012 (22955616)
	Phosphoglycerate mutase 1	Yes	*3'-UTR*	*Nature* 489:57; 2012 (22955616)
	Enolase 2	Yes	*3'-UTR, 5'UTR*	*Nature* 489:57; 2012 (22955616)
	Pyruvate kinase M1/2	Yes	*3'-UTR, 5'UTR, coding region*	*Nature* 489:57; 2012 (22955616)
HuR	Aldolase C	Yes	*5'UTR, coding region*	*Genome Biol* 15:R2; 2014 (24393468)
	Glucose transporter 1	Yes	*3'-UTR, 5'UTR, coding region*	*Nat Methods* 15:559; 2011 (21572407)<br>*Mol Cell* 43:327; 2011 (21723170)<br>*Mol Cell* 43:340; 2011 (21723171)<br>*J Biol Chem* 286:37063; 2011 (21890634)<br>*Genome Biol* 15:R2; 2014 (24393468)
PCBP1	Glucose transporter 1	Yes	*Exon; intron*	*bioRxiv* 635888; 2019 (GEO GSE131210)
	Glucose-6-phosphate isomerase	Yes	*Exon (majority); intron*	*bioRxiv* 635888; 2019 (GEO GSE131210)
	Aldolase A	No	-	*bioRxiv* 635888; 2019 (GEO GSE131210)
	Aldolase C	Yes	*Exon*	*bioRxiv* 635888; 2019 (GEO GSE131210)
	Glyceraldehyde 3-phosphate dehydrogenase	Yes	*Exon (majority); intron*	*bioRxiv* 635888; 2019 (GEO GSE131210)
	Phosphoglycerate kinase 1	No	-	*bioRxiv* 635888; 2019 (GEO GSE131210)
	Phosphoglycerate mutase 1	Yes	*Exon; intron*	*bioRxiv* 635888; 2019 (GEO GSE131210)
	Enolase 1	Yes	*Exon (majority); intron*	*bioRxiv* 635888; 2019 (GEO GSE131210)

Supplementary Table 2. Evidence for direct interactions between hypoxia-activated RBPs and mRNAs of glycolytic effectors from publicly available CLIP sequencing data, including the use of the starBase algorithm and database.

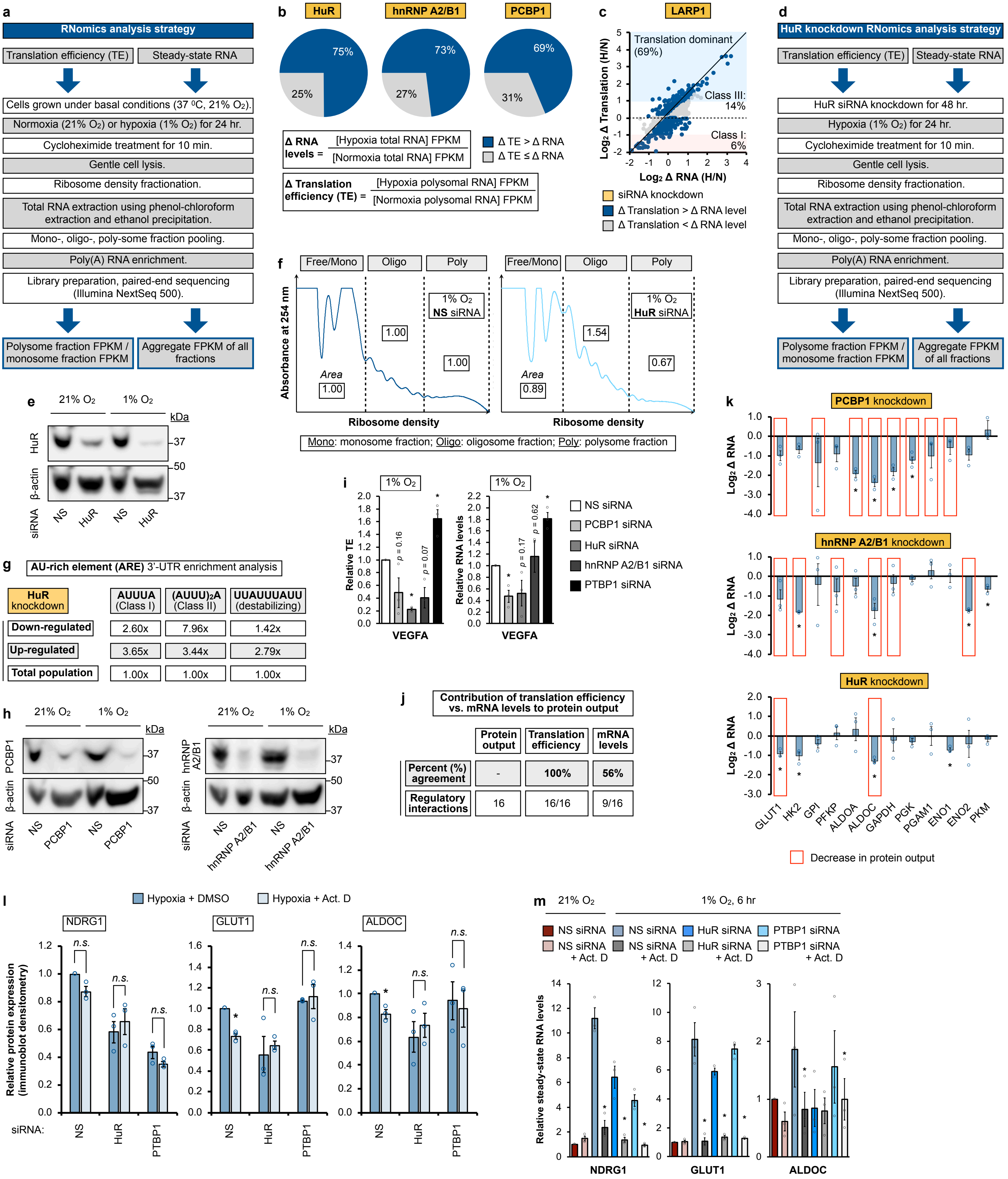
a
RNomics analysis strategy
Translation efficiency (TE)
Steady-state RNA
Cells grown under basal conditions (37 ºC, 21% O2).
Normoxia (21% O2) or hypoxia (1% O2) for 24 hr.
Cycloheximide treatment for 10 min.
Gentle cell lysis.
Ribosome density fractionation.
Total RNA extraction using phenol-chloroform extraction and ethanol precipitation.
Mono-, oligo-, poly-some fraction pooling.
Poly(A) RNA enrichment.
Library preparation, paired-end sequencing (Illumina NextSeq 500).
Polysome fraction FPKM / monosome fraction FPKM
Aggregate FPKM of all fractions
b
HuR
hnRNP A2/B1
PCBP1
75%
25%
73%
27%
69%
31%
Δ RNA levels = [Hypoxia total RNA] FPKM / [Normoxia total RNA] FPKM
Δ Translation efficiency (TE) = [Hypoxia polysomal RNA] FPKM / [Normoxia polysomal RNA] FPKM
Δ TE > Δ RNA
Δ TE ≤ Δ RNA
c
LARP1
Translation dominant (69%)
Class III: 14%
Class I: 6%
Log2 Δ Translation (H/N)
Log2 Δ RNA (H/N)
siRNA knockdown
Δ Translation > Δ RNA level
Δ Translation < Δ RNA level
d
HuR knockdown RNomics analysis strategy
Translation efficiency (TE)
Steady-state RNA
HuR siRNA knockdown for 48 hr.
Hypoxia (1% O2) for 24 hr.
Cycloheximide treatment for 10 min.
Gentle cell lysis.
Ribosome density fractionation.
Total RNA extraction using phenol-chloroform extraction and ethanol precipitation.
Mono-, oligo-, poly-some fraction pooling.
Poly(A) RNA enrichment.
Library preparation, paired-end sequencing (Illumina NextSeq 500).
Polysome fraction FPKM / monosome fraction FPKM
Aggregate FPKM of all fractions
e
21% O2
1% O2
kDa
HuR
β-actin
siRNA
NS
HuR
NS
HuR
f
Free/Mono
Oligo
Poly
1% O2 NS siRNA
1% O2 HuR siRNA
Area
1.00
1.00
1.00
1.54
0.67
0.89
Absorbance at 254 nm
Ribosome density
Mono: monosome fraction; Oligo: oligosome fraction; Poly: polysome fraction
g
AU-rich element (ARE) 3′-UTR enrichment analysis
HuR knockdown | AUUUA (Class I) | (AUUU)2A (Class II) | UUAUUUAUU (destabilizing)
Down-regulated | 2.60x | 7.96x | 1.42x
Up-regulated | 3.65x | 3.44x | 2.79x
Total population | 1.00x | 1.00x | 1.00x
h
21% O2
1% O2
kDa
PCBP1
β-actin
hnRNP A2/B1
siRNA
NS
PCBP1
hnRNP A2/B1
i
1% O2
Relative TE
Relative RNA levels
VEGFA
p = 0.16
p = 0.07
p = 0.17
p = 0.62
NS siRNA
PCBP1 siRNA
HuR siRNA
hnRNP A2/B1 siRNA
PTBP1 siRNA
j
Contribution of translation efficiency vs. mRNA levels to protein output
| Protein output | Translation efficiency | mRNA levels
Percent (%) agreement | - | 100% | 56%
Regulatory interactions | 16 | 16/16 | 9/16
k
PCBP1 knockdown
hnRNP A2/B1 knockdown
HuR knockdown
Log2 Δ RNA
GLUT1
HK2
GPI
PFKP
ALDOA
ALDOC
GAPDH
PGK
PGAM1
ENO1
ENO2
PKM
Decrease in protein output
l
Hypoxia + DMSO
Hypoxia + Act. D
NDRG1
GLUT1
ALDOC
n.s.
Relative protein expression (immunoblot densitometry)
siRNA:
NS
HuR
PTBP1
m
21% O2
1% O2, 6 hr
NS siRNA
HuR siRNA
PTBP1 siRNA
NS siRNA + Act. D
HuR siRNA + Act. D
PTBP1 siRNA + Act. D
Relative steady-state RNA levels
NDRG1
GLUT1
ALDOC

Supplementary Figure 5. (**a**) Schematic of oxygen-dependent RNomics analysis strategy. (**b**) Percentage of RBP-dependent targets that exhibit a larger translation efficiency fold change compared to RNA level modification. (**c**) Global analysis of hypoxia-induced translational versus RNA-level changes in U87MG using RNA sequencing of ribosome density fractions for LARP1-dependent targets. Δ translation and Δ RNA are calculated based on polysomal RNA abundance and total aggregate RNA across all fractions, respectively. (**d**) Schematic of HuR-dependent RNomics analysis strategy. (**e**) Representative control immunoblots of U87MG for HuR-dependent RNA sequencing analysis. Three independent experiments (n=3) were performed with similar results. (**f**) Ribosome density profiles of U87MG treated with HuR-specific and non-silencing (NS) control siRNAs (for 48 hr prior to following experimentation). Area: Area under curve, relative to corresponding normoxic fraction. Three independent experiments (n=3) were performed with similar results. (**g**) 3'-UTR ARE enrichment analysis of hypoxic, HuR-knockdown RNA sequencing results. (**h**) Representative control immunoblots of PCBP1- and hnRNP A2/B1-dependent qRT-PCR analysis of ribosome density fractions. Three independent experiments (n=3) were performed with similar results. NS: Non-silencing. (**i**) qRT-PCR measurements of changes in hypoxic TE (left panel) and steady-state mRNA levels (right panel) of VEGFA in U87MG treated with indicated siRNAs. NS: Non-silencing. Asterisk denotes statistical significance calculated using two-sided Student's t-tests compared to NS control. Exact p values: TE: HuR siRNA (p = 0.0008), PTBP1 siRNA (p = 0.013); Total RNA: PCBP1 siRNA (p = 0.038), PTBP1 siRNA (p = 0.016). Data represent mean±SEM (error bars) of three independent experiments (n=3). (**j**) Percent agreement/predictive value of translation efficiency versus steady-state mRNA levels (qRT-PCR measurements of three independent experiments) and protein output (TMT-pSILAC analysis of three independent experiments pooled into a single sample for measurement). (**k**) Hypoxic steady-state mRNA level regulation of glycolytic effectors by the hypoxia-adaptive RBPs PCBP1, hnRNP A2/B1, and HuR, as determined by qRT-PCR of ribosome density fractions. Asterisk denotes statistical significance calculated using two-sided Student's t-tests compared to non-silencing control. Exact p values: PCBP1 siRNA: ALDOA (p = 0.007), ALDOC (p = 0.006), GAPDH (p = 0.013), PGK (p = 0.015); hnRNP A2/B1 siRNA: HK2 (p = 0.0001), ALDOC (p = 0.043), ENO2 (p = 0.0007), PKM (p = 0.034); HuR siRNA: GLUT1 (p = 0.01), HK2 (p = 0.038), ALDOC (p = 0.002), ENO1 (p = 0.015). Data represent mean±SEM (error bars) of three independent experiments (n=3). Red box: observed decrease in protein output as determined by TMT-pSILAC. (**l**) Densitometry of immunoblots in Fig. 4g. Asterisk denotes statistical significance calculated using two-sided Student's t-tests compared to corresponding hypoxia + DMSO control. Exact p values: GLUT1 NS siRNA (p =0.0096); ALDOC NS siRNA(p = 0.042). Data represent mean±SEM (error bars) of three independent experiments (n=3). (**m**) Steady-state mRNA level measurements by qRT-PCR of normoxic and hypoxic (6 hr) U87MG treated with actinomycin D (Act. D) or vehicle DMSO. NS: Non-silencing. Asterisk denotes statistical significance calculated using two-sided Student's t-tests compared to NS control. Exact p values: NDRG1 (1% O_2): NS siRNA (p = 0.0017), HuR siRNA (p = 0.0005), PTBP1 siRNA (p = 0.0007); GLUT1 (1% O_2): NS siRNA (p = 0.0002), HuR siRNA (p = 0.0005), PTBP1 siRNA (p = 6.54e-05); ALDOC (1% O_2): NS siRNA (p = 0.012), PTBP1 siRNA (p = 0.0078). Data represent mean±SEM (error bars) of three independent experiments (n=3). Source data are provided as a Source Data file.

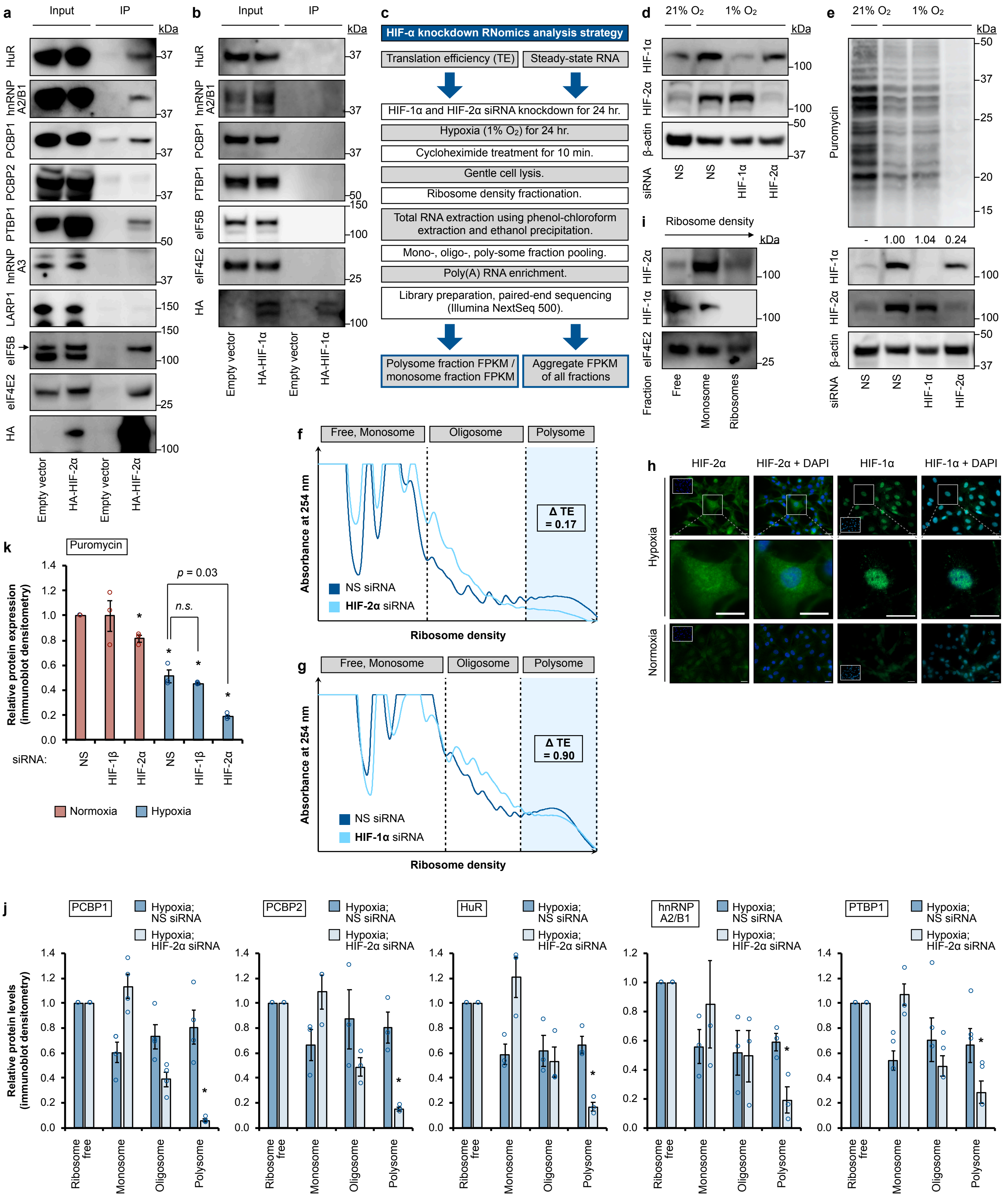

a
Input
IP
kDa
HuR
hnRNP A2/B1
PCBP1
PCBP2
PTBP1
hnRNP A3
LARP1
eIF5B
eIF4E2
HA
Empty vector
HA-HIF-2α
Empty vector
HA-HIF-2α
b
Input
IP
kDa
HuR
hnRNP A2/B1
PCBP1
PTBP1
eIF5B
eIF4E2
HA
Empty vector
HA-HIF-1α
Empty vector
HA-HIF-1α
c
HIF-α knockdown RNomics analysis strategy
Translation efficiency (TE)
Steady-state RNA
HIF-1α and HIF-2α siRNA knockdown for 24 hr.
Hypoxia (1% O2) for 24 hr.
Cycloheximide treatment for 10 min.
Gentle cell lysis.
Ribosome density fractionation.
Total RNA extraction using phenol-chloroform extraction and ethanol precipitation.
Mono-, oligo-, poly-some fraction pooling.
Poly(A) RNA enrichment.
Library preparation, paired-end sequencing (Illumina NextSeq 500).
Polysome fraction FPKM / monosome fraction FPKM
Aggregate FPKM of all fractions
d
21% O2
1% O2
kDa
HIF-1α
HIF-2α
β-actin
siRNA
NS
NS
HIF-1α
HIF-2α
e
21% O2
1% O2
kDa
Puromycin
- 1.00 1.04 0.24
HIF-1α
HIF-2α
β-actin
siRNA
NS
NS
HIF-1α
HIF-2α
i
Ribosome density
kDa
HIF-2α
HIF-1α
eIF4E2
Fraction
Free
Monosome
Ribosomes
f
Free, Monosome
Oligosome
Polysome
Absorbance at 254 nm
Δ TE = 0.17
NS siRNA
HIF-2α siRNA
Ribosome density
g
Free, Monosome
Oligosome
Polysome
Absorbance at 254 nm
Δ TE = 0.90
NS siRNA
HIF-1α siRNA
Ribosome density
h
HIF-2α
HIF-2α + DAPI
HIF-1α
HIF-1α + DAPI
Hypoxia
Normoxia
k
Puromycin
Relative protein expression (immunoblot densitometry)
p = 0.03
n.s.
siRNA:
NS
HIF-1β
HIF-2α
NS
HIF-1β
HIF-2α
Normoxia
Hypoxia
j
PCBP1
PCBP2
HuR
hnRNP A2/B1
PTBP1
Hypoxia; NS siRNA
Hypoxia; HIF-2α siRNA
Relative protein levels (immunoblot densitometry)
Ribosome free
Monosome
Oligosome
Polysome

Supplementary Figure 6. Representative immunoblots of (**a**) HA-HIF-2α and (**b**) HA-HIF-1α co-immunoprecipitations in U87MG. Three independent experiments (n=3) were performed with similar results. (**c**) Schematic of HIF-α RNomics analysis strategy. (**d**) Representative control immunoblots of U87MG for HIF-1α and HIF-2α-dependent RNA sequencing analysis of ribosome density fractions. Three independent experiments (n=3) were performed with similar results. (**e**) Representative immunoblots of U87MG treated with indicated siRNAs. Puromycin incorporation was used as a measure of global translational intensity. NS: Non-silencing. Quantitation represents mean of three independent experiments (n=3). Ribosome density profiles of U87MG treated with (**f**) HIF-2α- and (**g**) HIF-1α-specific versus non-silencing (NS) control siRNAs. Δ translation efficiency (TE) was determined based on area under curve ratios of polysome/free, monosome in HIF-α-specific versus NS siRNA samples. (**h**) Representative immunocytochemistry images of endogenous HIF-2α and HIF-1α in normoxic and hypoxic U87MG (n=10 fields over three independent experiments). Scale bar: 20 μm. (**i**) Representative immunoblots of hypoxic U87MG ribosome density fractions. Three independent experiments (n=3) were performed with similar results. (**j**) Densitometry of immunoblots in Fig. 5f. Asterisk denotes statistical significance calculated using two-sided Student's t-tests compared to NS control. Exact p values: PCBP1 ($p = 0.021$), PCBP2 ($p = 0.038$), HuR ($p = 0.041$), hnRNP A2/B1 ($p = 0.0093$), PTBP1 ($p = 0.049$). Data represent mean±SEM (error bars) of three independent experiments (n=3). (**k**) Densitometry of immunoblots in Fig. 5h. Asterisk denotes statistical significance calculated using two-sided Student's t-tests compared to normoxia NS control. Exact p values: Normoxia: HIF-2α siRNA ($p = 0.018$); Hypoxia: NS siRNA ($p = 0.01$), HIF-1β siRNA ($p = 9.63e-05$), HIF-2α siRNA ($p = 0.00029$). Data represent mean±SEM (error bars) of three independent experiments (n=3). Source data are provided as a Source Data file.

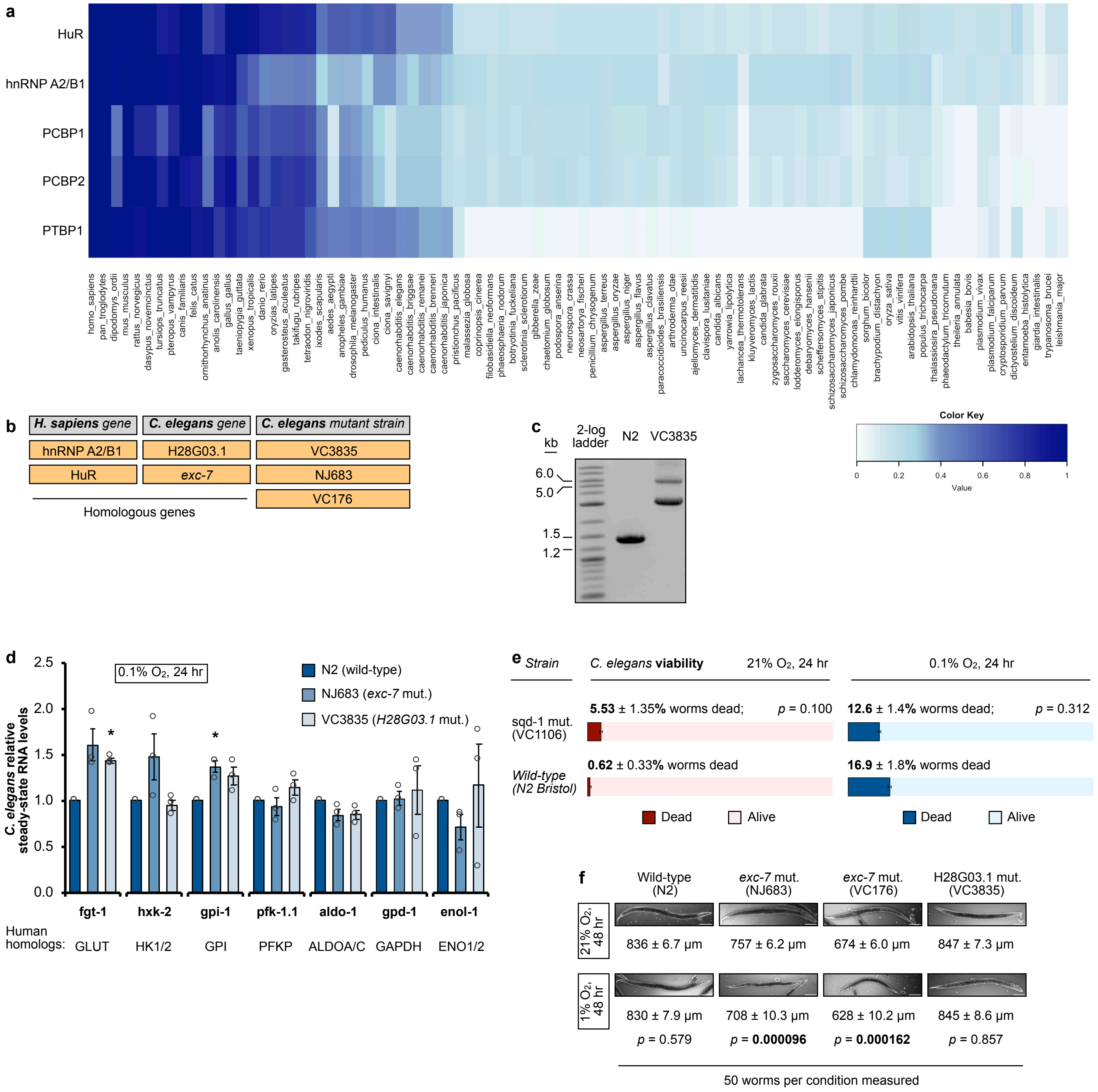

Supplementary Figure 7. (**a**) Normalized phylogenetic profiling using the PhyloGene algorithm across species. (**b**) *C. elegans* homologs of HuR and hnRNP A2/B1 and strains carrying deletion mutations. (**c**) Representative PCR genotyping of the H28G03.1 mutation in the VC3835 strain. This results was confirmed in three independent experiments (n=3). (**d**) Total steady-state mRNA levels of glycolytic effectors in hypoxic (0.1% O_2) *C. elegans* that contain wild-type (N2) or mutant homologs of HuR (exc-7, strain NJ683) or hnRNP A2/B1 (H28G03.1, strain VC3835), as measured by qRT-PCR. Asterisk denotes statistical significance calculated using two-sided Student's t-tests compared to wild-type control. Exact p values: NJ683: gpi-1 (p = 0.032); VC3835: fgt-1 (p = 0.0032). Data represent mean±SEM (error bars) of three independent experiments (n=3). (**e**) Measurements of normoxic and hypoxic death for adult (Day 1 at the start of treatment) *C. elegans* that contain wild-type (N2) or mutant sqd-1, a paralog of H28G03.1 (strain VC1106). Data represent mean±SEM (error bars) of three independent experiments (n=3; total number of worms for N2, VC1106: 703, 270, respectively). Statistical significance was calculated using two-sided Student's t-tests compared to wild-type control. Exact p values are indicated in the figure. (**f**) Representative images depicting differences in body length of *C. elegans* strains (L1 larvae at the start of treatment) under normoxic and hypoxic conditions. Scale bar: 100 μm. Measurements represent mean±SEM (n=50 worms). Statistical significance was calculated using two-sided Student's t-tests compared to corresponding normoxia control. Exact p values are indicated in the figure. Source data are provided as a Source Data file.
