# Supplementary material for: A network of RNA-binding proteins controls translation efficiency to activate anaerobic metabolism
Source: Nat Commun. 2020 May 29;11:2677. doi: 10.1038/s41467-020-16504-1 (PMC7260222; doi:10.1038/s41467-020-16504-1)
Supplement: Supplementary file 2 — Reporting Summary [file 41467_2020_16504_MOESM2_ESM.pdf]

nature research

Corresponding author(s): Stephen Lee

Last updated by author(s): Apr 17, 2020

Reporting Summary

Nature Research wishes to improve the reproducibility of the work that we publish. This form provides structure for consistency and transparency in reporting. For further information on Nature Research policies, see Authors & Referees and the Editorial Policy Checklist.

Statistics

For all statistical analyses, confirm that the following items are present in the figure legend, table legend, main text, or Methods section.

n/a	Confirmed	
☐	☒	The exact sample size (*n*) for each experimental group/condition, given as a discrete number and unit of measurement
☐	☒	A statement on whether measurements were taken from distinct samples or whether the same sample was measured repeatedly
☐	☒	The statistical test(s) used AND whether they are one- or two-sided<br>*Only common tests should be described solely by name; describe more complex techniques in the Methods section.*
☐	☒	A description of all covariates tested
☐	☒	A description of any assumptions or corrections, such as tests of normality and adjustment for multiple comparisons
☐	☒	A full description of the statistical parameters including central tendency (e.g. means) or other basic estimates (e.g. regression coefficient) AND variation (e.g. standard deviation) or associated estimates of uncertainty (e.g. confidence intervals)
☒	☐	For null hypothesis testing, the test statistic (e.g. F, t, r) with confidence intervals, effect sizes, degrees of freedom and P value noted<br>*Give P values as exact values whenever suitable.*
☒	☐	For Bayesian analysis, information on the choice of priors and Markov chain Monte Carlo settings
☒	☐	For hierarchical and complex designs, identification of the appropriate level for tests and full reporting of outcomes
☒	☐	Estimates of effect sizes (e.g. Cohen's *d*, Pearson's *r*), indicating how they were calculated

Our web collection on statistics for biologists contains articles on many of the points above.

Software and code

Policy information about availability of computer code

Data collection	Mass spectrometry: Data were collected using an Orbitrap Fusion Lumos Tribrid mass spectrometer (ThermoFisher Scientific) (TMT-pSILAC) or an LTQ-Orbitrap Elite hybrid mass spectrometer (Thermo-Fisher) (MATRIX).<br>RNA sequencing: Illumina NextSeq 500 generated paired-end FASTQ files, that were stored and downloaded from Illumina Basespace.
Data analysis	Mass spectrometry: MS raw files were processed using Proteome Discoverer 2.2 (Thermo Fisher Scientific) (TMT-pSILAC). MS raw files were processed using PEAKS software (v8.5, Bioinformatics Solutions Inc.) (MATRIX).<br>RNA sequencing: Reads were quality-checked using Cutadapt (v1.15), aligned to human reference genome GRCh38 (release 27) with STAR (v2.3) and annotated/quantified with Cufflinks (v2.2.1) to determine FPKM values for reference genome-annotated genes.<br>Immunoblot densitometry was performed using ImageJ software (v1.5, NIH).<br>GO enrichment analysis was performed using the online Database for Annotation, Visualization and Integrated Discovery (DAVID) bioinformatics resource (v6.8, https://david.ncifcrf.gov).

For manuscripts utilizing custom algorithms or software that are central to the research but not yet described in published literature, software must be made available to editors/reviewers. We strongly encourage code deposition in a community repository (e.g. GitHub). See the Nature Research guidelines for submitting code & software for further information.

Data

Policy information about availability of data

All manuscripts must include a data availability statement. This statement should provide the following information, where applicable:

- Accession codes, unique identifiers, or web links for publicly available datasets
- A list of figures that have associated raw data
- A description of any restrictions on data availability

MS datasets are available via the ProteomeXchange accessions: PXD011979, PXD006799. MS data was searched against the Human Uniprot Database (https://www.uniprot.org). RNA sequencing datasets are available via the NCBI GEO accessions: GSE128541, GSE128547, GSE128555. The source data underlying Figures 1c-h, 2b-e, 2g, 3e, 3f, 4a, 4d, 5a, 5f-h, 6c-g, and Supplementary Figures 1c, 1e, 1g, 1i, 2b, 2e-h, 3c, 3g, 4d, 4e, 4g, 5l, 5m, 6j, 6k, 7d are provided as a Source Data file.

Proteomic data: PXD011979. Reviewer token: Username: reviewer50001@ebi.ac.uk; Password: ch2nnZKf. PXD006799.
MS data were searched against the Human Uniprot Database (https://www.uniprot.org) consisting of only reviewed entries using the Sequest HT and MS Amanda 2.0 search engines.
RNA-seq data: GSE128541, GSE128547, GSE128555. Reviewer tokens: cdwvqwsqpdqzbgh, ojetaykcfvcrvaj, mfwnakkmhzsfjqp, respectively.

Field-specific reporting

Please select the one below that is the best fit for your research. If you are not sure, read the appropriate sections before making your selection.

☒ Life sciences ☐ Behavioural & social sciences ☐ Ecological, evolutionary & environmental sciences

For a reference copy of the document with all sections, see nature.com/documents/nr-reporting-summary-flat.pdf

Life sciences study design

All studies must disclose on these points even when the disclosure is negative.

Sample size	No sample size calculations were performed,.We performed a minimum of three independent experiments, which sufficiently confirmed reproducibility, and enabled statistical analyses.
Data exclusions	No data were excluded.
Replication	Experiments were performed at least three independent times, and independent approaches were performed to ensure the reproducibility of experimental findings. Exact number of independent experiments ('n') are provided in figure legends.
Randomization	Randomization was not performed in our studies per se. For each experiment, cell lines and genetically identical organisms used were derived from the same parent population/passage.
Blinding	Blinding was not performed in our studies, as our pre-determined protocols required research personnel to have knowledge of treatment conditions. Objective, quantitative approaches were used to gather and analyze experimental data.

Reporting for specific materials, systems and methods

We require information from authors about some types of materials, experimental systems and methods used in many studies. Here, indicate whether each material, system or method listed is relevant to your study. If you are not sure if a list item applies to your research, read the appropriate section before selecting a response.

Materials & experimental systems

n/a	Involved in the study	
☐	☒	Antibodies
☐	☒	Eukaryotic cell lines
☒	☐	Palaeontology
☐	☒	Animals and other organisms
☒	☐	Human research participants
☒	☐	Clinical data

Methods

n/a	Involved in the study	
☒	☐	ChIP-seq
☒	☐	Flow cytometry
☒	☐	MRI-based neuroimaging

Antibodies

Antibodies used	PCBP1 (Cell Signaling, #8534S; 1:1000 dilution); PCBP2 (Abnova, #H00005094; 1:1000 dilution); HuR (Cell Signaling, #12582S; 1:1000 dilution); hnRNP A2/B1 (2A2) (Proteintech, #14183-1-AP; 1:1000 dilution); PTBP1 (Cell Signaling, #8776S; 1:1000 dilution); LARP1 (Bethyl Laboratories, #A302-087A; 1:1000 dilution); hnRNP A3 (Proteintech, #25142-1-AP; 1:1000 dilution); hnRNP C (Santa Cruz Biotechnology, #sc-32308; 1:1000 dilution); NDRG1 (Abcam, #ab37897; 1:2000 dilution); GLUT1 (Novus Biologicals, # NB110-39113; 1:1000 dilution); ALDOC (Proteintech, #14884-1-AP; 1:1000 dilution); F3 (Abcam, #ab104513; 1:1000 dilution); PAI-1 (Proteintech, #13801-1-AP; 1:1000 dilution); HIF-1α (Novus Biologicals, #NB100-105; 1:1000 dilution for immunoblot); HIF-1α (Novus Biologicals, #AF1935; 1:100 dilution for immunocytochemistry); HIF-2α (Novus Biologicals, #NB100-122; 1:1000 dilution); HIF-1β (Novus Biologicals, #NB100-110; 1:1000 dilution); puromycin (3RH11) (Kerafast, #EQ0001; 1:1000 dilution); eIF5B (Santa Cruz Biotechnology, #393564; 1:1000 dilution); β-actin (C4) (Santa Cruz Biotechnology, #sc-47778; 1:5000 dilution); Rabbit IgG isotype control (Novus Biologicals, #NB810-56910; 5 µg per immunoprecipitation reaction); Goat anti-rabbit IgG secondary, HRP-conjugated (Novus Biologicals, #NB7160; 1:10000 dilution for immunoblot); Goat anti-mouse IgG secondary, HRP-conjugated (Proteintech, #SA00001-1; 1:10000 dilution for immunoblot); Donkey anti-goat IgG secondary, HRP-conjugated (Novus Biologicals, #NB7357; 1:10000 dilution for immunoblot); Goat anti-rabbit IgG secondary, Alexa Fluor 488-conjugated (ThermoFisher, #A-11008; 1:500 dilution for immunocytochemistry); Donkey anti-goat IgG secondary, Alexa Fluor 488-conjugated (ThermoFisher, #A-11055; 1:500 dilution for immunocytochemistry).
Validation	Target specificity in human samples were verified by immunoblot for the following primary antibodies in our study by RNAi-mediated silencing: PCBP1 (Cell Signaling, #8534S; 1:1000 dilution); PCBP2 (Abnova, #H00005094; 1:1000 dilution); HuR (Cell

Signaling, #12582S; 1:1000 dilution); hnRNP A2/B1 (2A2) (Proteintech, #14183-1-AP; 1:1000 dilution); PTBP1 (Cell Signaling, #8776S; 1:1000 dilution); LARP1 (Bethyl Laboratories, #A302-087A; 1:1000 dilution); hnRNP A3 (Proteintech, #25142-1-AP; 1:1000 dilution); HIF-1α (Novus Biologicals, #NB100-105; 1:1000 dilution for immunoblot); HIF-2α (Novus Biologicals, #NB100-122; 1:1000 dilution); HIF-1β (Novus Biologicals, #NB100-110; 1:1000 dilution).
Empirical information on antibody target specificity in human samples for immunoblot applications are available on corresponding manufacturer websites.

Eukaryotic cell lines

Policy information about cell lines

Cell line source(s)	All cell lines (U87MG, PC3, A549, HCT116, NIH/3T3) were purchased from the American Type Culture Collection.
Authentication	Cell lines were authenticated by cytogenetic analysis, STR profiling, and isoenzyme profiling by the manufacturer.
Mycoplasma contamination	All cell lines tested negative for mycoplasma contamination.
Commonly misidentified lines (See ICLAC register)	No commonly misidentified cell lines were used.

Animals and other organisms

Policy information about studies involving animals; ARRIVE guidelines recommended for reporting animal research

Laboratory animals	C. elegans: N2 Bristol (wild-type); VC3835: H28G03.1(gk3802); NJ683: exc-7(rh252); VC176: exc-7(ok370). C. elegans are hermaphrodites, used for experiments 1 day past larval stage 4.
Wild animals	Our study did not involve wild animals.
Field-collected samples	Our study did not involve samples collected from the field.
Ethics oversight	Study protocols were approved by the University of Miami Institutional Animal Care & Use Committee.

Note that full information on the approval of the study protocol must also be provided in the manuscript.
